# Poverty and suicide research in low- and middle-income countries: systematic mapping of literature published in English and a proposed research agenda

**DOI:** 10.1017/gmh.2016.27

**Published:** 2016-12-13

**Authors:** J. Bantjes, V. Iemmi, E. Coast, K. Channer, T. Leone, D. McDaid, A. Palfreyman, B. Stephens, C. Lund

**Affiliations:** 1 Department of Psychology, Stellenbosch University, Private Bag X1, Matieland 7602, Cape Town, South Africa; 2 Personal Social Services Research Unit, London School of Economics and Political Science, Houghton Street, London WC2A 2AE, UK; 3 LSE Health, London School of Economics and Political Science, Houghton Street, London WC2A 2AE, UK; 4 Peterborough Child Development Centre, City Care Centre, Thorpe Road, Peterborough PE3 6DB, UK; 5 Department of Psychiatry and Mental Health, Alan J Flisher Centre for Public Mental Health, University of Cape Town, 46 Sawkins Road, Rondebosch 7700, Cape Town, South Africa; 6 Centre for Global Mental Health, Institute of Psychiatry, Psychology and Neuroscience, King's College London, London, UK

**Keywords:** Low- and middle-income countries, poverty, suicide, suicide prevention, systematic mapping

## Abstract

Approximately 75% of suicides occur in low- and middle-income countries (LMICs) where rates of poverty are high. Evidence suggests a relationship between economic variables and suicidal behaviour. To plan effective suicide prevention interventions in LMICs we need to understand the relationship between poverty and suicidal behaviour and how contextual factors may mediate this relationship. We conducted a systematic mapping of the English literature on poverty and suicidal behaviour in LMICs, to provide an overview of what is known about this topic, highlight gaps in literature, and consider the implications of current knowledge for research and policy. Eleven databases were searched using a combination of key words for suicidal ideation and behaviours, poverty and LMICs to identify articles published in English between January 2004 and April 2014. Narrative analysis was performed for the 84 studies meeting inclusion criteria. Most English studies in this area come from South Asia and Middle, East and North Africa, with a relative dearth of studies from countries in Sub-Saharan Africa. Most of the available evidence comes from upper middle-income countries; only 6% of studies come from low-income countries. Most studies focused on poverty measures such as unemployment and economic status, while neglecting dimensions such as debt, relative and absolute poverty, and support from welfare systems. Most studies are conducted within a risk-factor paradigm and employ descriptive statistics thus providing little insight into the nature of the relationship. More robust evidence is needed in this area, with theory-driven studies focussing on a wider range of poverty dimensions, and employing more sophisticated statistical methods.

Suicide prevention has been highlighted as a global public mental health issue by the recent World Health Organisation report on suicide (WHO, 2014) and the United Nations proposal to include suicide rates as a key indicator for target 3.4 of the Sustainable Development Goals. Suicide is the tenth leading cause of death globally (Hawton & van Heeringen, 2009) and it is estimated that as many as 804 000 suicide deaths occurred worldwide in 2012 (WHO, 2014). It is estimated that rates of non-fatal suicidal behaviour are 20 to 30 times more common than completed suicides (Wasserman, 2001). In Ireland for instance in 2013 rates of self-harm for men were 182 per 100 000 and for women 217 per 100 000, which is higher than the suicide rates of 17.4 and 3.9 per 100 000, respectively (Griffin *et al.* 2015). For every suicide attempt an estimated 10 people experience suicidal ideation (Borges *et al.* 2010). As many as 75.5% of suicides occur in low- and middle-income countries (LMICs) (WHO, 2014). A large body of evidence documents the psychiatric risk factors for suicidal behaviours (Hawton *et al*. 2005*a*, *b*; Krysinska & Lester, 2010). A growing body of literature documents the relationship between suicide and socio-economic variables, such as poverty, financial crisis, indebtedness and unemployment (Brinkmann, 2009; Fliege *et al.* 2009; Platt, 2011; Chan, 2013; Coope *et al.* 2014; Haw *et al.* 2015). The recently published systematic review of 37 studies utilising multivariate analysis of the relationship between poverty and suicidal ideation and behaviours in LMICs, is a further example of literature in this field (Iemmi *et al.*, 2016). Understanding relationships between poverty and suicide is important for suicide prevention, especially in LMICs where rates of poverty and suicide are high and where the economic costs of suicidal behaviour are substantial. It is within this context that we conducted a systematic mapping of the literature published in English on poverty and suicidal behaviour in LMICs. Our intention was to consider critically what has hitherto been the focus of research on poverty and suicidal behaviour and identify possible future directions for research. We focused on methodological issues (such as measurement, study design, methods of statistical analysis and theoretical frameworks) employed in the published English literature, with a view to making suggestions for how future research in this field might be strengthened in order to make meaningful contributions to suicide prevention in LMICs.

There are compelling reasons for improving our understanding of how poverty and suicidal behaviour affect each other in LMICs. Better understanding the socio-economic determinants of suicidal behaviour could assist policy makers to develop population-level interventions. Without an understanding of the social and economic determinants of suicidal behaviour, health systems in LMICs are unlikely to provide effective suicide prevention interventions or to have the human resources to support those who engage in suicidal behaviour. In LMICs there are <0.5 psychiatrists per 100 000 population, compared with 6.6 per 100 000 in high-income countries (HICs) (WHO, 2014); highlighting the need to consider broad social determinants of suicidal behaviour in LMICs rather than only focusing on psychiatric causes and individuals already identified as suicidal who are in contact with the health care system. This requires expanding approaches such as the ‘four level approach’ of the European Alliance Against Depression intervention, which focuses on population wide interventions aimed at recognising risk and promoting access to psychiatric care (Hegerl *et al.* 2009), but pays insufficient attention to the distal socio-economic determinants of suicidal behaviour, such as poverty.

## Defining suicidal behaviours

Clear and explicit definitions of concepts are necessary for accurate measurement, effective communication of research findings and the application of findings to suicide prevention. There is, however, no universal set of terms and definitions, which are neither consistently used in suicidology, nor is there an agreed taxonomy for classifying the complete spectrum of suicide-related behaviours (Silverman *et al.* 2007). This problem is further compounded by the fact that suicidology is a multidisciplinary field, which attracts scholars from a range of disciplines, each with their own terminology and theoretical assumptions (Silverman, 2011). Enduring debate about nomenclature in suicide research has perpetuated the use of a variety of terms in the published literature to denote the same behaviours and the inconsistent use of terms (Silverman, 2006). This lack of precision presents particular challenges for researchers, policy makers and clinicians who seek to integrate existing suicide research, compare findings across different studies or apply research findings to clinical practice and suicide prevention.

For the purpose of this systematic mapping, we considered the full spectrum of suicidal phenomena (including suicidal ideations, communications, self-injurious behaviours and suicide deaths). The decision to include the full spectrum of suicidal phenomena was driven by: (1) our desire to create a broad overview of the field; and (2) by literature which suggests suicidal ideation and non-fatal suicidal behaviour are both predictive of completed suicide, which implies that suicide prevention initiatives should focus on the entire spectrum of suicidal phenomena (WHO, 2014). However, we acknowledge that assumptions about clear links between different suicidal phenomena are contested; there are few studies, which compare suicidal ideation, planning and deaths from suicide in different national studies, and some authors have found no relationship between suicide and non-fatal suicidal ideation and behaviours (Weissman *et al*. 1999; Bertolote *et al.* 2005).

We have used the term ‘suicidal behaviour’ as it was used in the WHO suicide report to refer to the entire spectrum of suicidal phenomena; ‘suicidal behaviour refers to a range of behaviours that include thinking about suicide (or ideation), planning for suicide, attempting suicide and suicide itself’ (WHO, 2014, p. 12).

In an effort to bring order to the research findings we have drawn the following distinctions between non-fatal suicidal behaviour and fatal suicidal behaviour:

- Non-fatal suicidal behaviour: We have taken the term non-fatal suicidal behaviour to denote suicidal ideation and behaviours directed towards intentionally ending one's life, but which do not result in death. We defined suicidal ideation as a cognitive occurrence characterised by thoughts of death and a desire to die; ‘suicidal ideation’ includes the wish or desire to die, thoughts of killing oneself without any intent to act on these, and intentions to kill oneself, including making suicide plans (Silverman *et al.* 2007). Non-fatal suicidal behaviours also include preparatory acts towards initiating a suicide plan, communicating suicidal intent, initiating a suicide plan, interrupted suicide attempts and suicide attempts. We have also considered any self-injurious behaviour with a non-fatal outcome, irrespective of whether death was intended (i.e. deliberate self-harm) to be a form of non-fatal suicidal behaviour.
- Fatal suicidal behaviour: The term fatal suicidal behaviour is taken to mean a death caused by deliberate self-injurious behaviour where there was non-zero intent to die (Posner *et al.* 2014). Fatal suicidal behaviour is synonymous with suicide.

## Socio-economic correlates of suicidal behaviour

Durkheim's sociological perspective established a tradition for considering socio-economic factors associated with suicide (Durkheim, 1897; Taylor, 1982). Durkheim provided evidence of correlations between suicide and measures of social integration and social regulation, defined as the moral and normative demands of society on individuals (Bearman, 1991). Durkheim theorised that equality in income and wealth (affluence or poverty) was protective against suicide; he argued that income inequality threatens social integration and results in anomie. Durkheim also speculated that poverty may be protective against suicide since affluence could lead people to believe they are dependant only on themselves, which may engender feelings of anomie and social disintegration. Durkheim's ideas, together with the literature documenting the impact of economic factors on health (Wilkinson & Pickett, 2006; Lund *et al.* 2010) have spurred a substantial body of research describing the relationship between suicidal behaviour and economic conditions, particularly in HICs.

Suicide has been associated with economic inequalities and economic shocks, both rapid booms and recessions (McDaid & Kennelly, 2009). The impact of unemployment on suicide in HICs has been extensively investigated in the wake of global economic crises. An Australian study reported higher rates of suicide following the 2006–2008 economic crisis (Milner *et al.* 2014). Following the 1997–1998 Asian economic crisis, a positive association was found between suicide and unemployment and gross domestic product (GDP) contraction in Japan, South Korea and Hong Kong, but not in Taiwan or Singapore (Chang *et al.* 2009). A study of 54 HICs showed that increased rates of unemployment following the economic crisis were associated with increases in suicide rates, although the strength of the association varied by gender and region (Chang *et al.* 2013). In the decade following the 1997–1998 Asian economic crisis, suicide rates in Korea rose at a significantly greater rate in the poorest sections of society left behind during the economic recovery (Hong *et al.* 2011). Similar trends were observed in Japan, Hong Kong and Korea (Chang *et al.* 2009). Suicide rates continued to rise in South Korea even after economic recovery; it was hypothesised that it takes much longer for the benefits of economic recovery to trickle down to the most economically vulnerable individuals leaving them susceptible to suicide even after economic recovery (Chan *et al.* 2014).

The strength of the association between suicide and poverty appears to be a function of a range of socio-demographic, geographic, and cultural factors. Studies focused on sub-groups of the population, such as the young, reveal associations that remain hidden when examining data at national level. For example, materially deprived rural men in Portugal were more vulnerable to suicide than the general population following the recent economic crises (Santana *et al.* 2015).

Socio-economic variables can also be protective against suicide. For example, the initial impact of the recent recession on suicide was buffered by the strength of family networks in Portugal and Spain (Wahlbeck & McDaid, 2012). Similarly, the availability of social welfare safety nets in Nordic countries exposed to economic uncertainty after the collapse of the Soviet Union appeared to be protective against suicide (Wahlbeck & McDaid, 2012).

Data on socio-economic correlates of non-fatal suicidal behaviour are more limited because of the lack of international databases for these phenomena and inconsistencies with recording and reporting (O'Connor *et al.* 2011). Data from 108 705 adults from 21 countries collected between 2001 and 2007 as part of the World Health Organization World Mental Health Surveys, found that lower income level and unemployment were risk factors for non-fatal suicidal behaviour in HICs and LMICs (Borges *et al.* 2010). A review by Platt & Hawton (2000) concluded that increased risk of deliberate self-harm was associated with being unemployed and inversely related to social class. There is some evidence from HICs to suggest that the strength of the association between non-fatal suicidal behaviour and social class may be a function of gender. For example, Platt *et al*. (1988) demonstrated a marked inverse relationship between social class and the incidence of hospital-treated non-fatal suicidal behaviours among men in the UK in the 1980s. A study of 2404 British adults found that suicidal ideation was associated with being unemployed (Gunnell *et al.* 2004).

## Economic consequences of suicide

Suicidal behaviour is not only a human tragedy; it has adverse economic consequences, particularly in resource scarce environments. There are economic costs associated with the morbidity and mortality caused by suicidal behaviour. Morbidity and mortality not only leads to a loss of productivity with financial implications for the individual and their family, but attending to suicidal individuals also requires resources. In this context it is significant that rates of suicide peak among the working-aged (WHO, 2014). Recent estimates from the USA suggest that the annual cost of suicidal behaviour in 2013 was $58.4 billion ($93.5 billion after adjusting for under-reporting) (Shepard *et al.* 2015). The USA analysis conservatively estimates a mean cost of just over $1 million per suicide; other estimates that include broader costs in Scotland, Ireland and New Zealand place the mean cost per suicide at $2.5, $2.3 and $2.1 million respectively (O'Dea & Tucker, 2005; Platt *et al.* 2006; Kennelly, 2007). Little is known about the costs of suicide in LMICs, even though LMICs account for the majority of the top ten suicide rates worldwide.

## Theoretical perspectives on suicidal behaviour

Advances in the field of suicidology have been hampered by the lack of theory development and the absence of widely accepted models of suicidal behaviour (Hawton, & Van Heeringen, 2000). The empirical and epidemiological research in suicidology has predominantly been conducted within a risk-factor paradigm, yielding a large number of studies describing correlates of suicidal behaviour. More recently a number of theories have been advanced describing how individual psychological characteristics and subjective experiences interact with socio-cultural and contextual factors to precipitate suicidal behaviour. For example, O'Connor's (2011) Integrated Motivational Volitional (IMV) model of suicidal behaviour, postulates that suicidal behaviour emerges as a result of feelings of entrapment; individuals who feel trapped by life circumstances and who perceive no other alternatives for escape employ suicidal behaviour as a means of seeking resolution. This idea is consistent with Shneidman's (1998) assertion that the common purpose of suicide is to seek a solution, and with Williams & Pollock's (2001) ‘arrested flight model’ of suicidal behaviour which asserts that suicidal behaviour results from the perception of being trapped with no possibility of rescue and no chance of escape. Joiner's (2005) interpersonal-psychological theory of suicide asserts that suicidal behaviour is a response to the psychological experience of ‘thwarted belonging’ and ‘perceived burdensomeness’ which are compounded by the experience of hopelessness. These theories prompt questions about how the subjective psychological experience of poverty might give rise to the experience of social disintegration or feelings of entrapment, thwarted belonging and perceived burdensomeness which in turn precipitate suicidal behaviour.

We have collected and analysed the data for this systematic mapping and presented our findings within the framework of a risk-factor model of suicidal behaviour. In other words we looked for literature in which poverty was investigated as a risk factor for suicidal behaviour (or vice versa) and arranged our findings by considering how different aspects of poverty (such as unemployment or indebtedness) might be considered risk factors for suicidal behaviour. In framing our recommendations for future research we have assumed that suicidal behaviour is a form of goal directed behaviour which is consciously initiated by individuals in response to their subjective psychological experience (perceptions, thoughts and feelings) of environmental factors (in this case poverty). In so doing we have aligned ourselves with the theoretical approaches proposed by Joiner (2005), O'Connor (2011), Shneidman (1998), and Williams & Pollock (2001). We have assumed that for suicide prevention it is not enough for researchers to simply investigate risk factors without also documenting individuals' subjective experiences of these factors.

## Methods

Our aim was to provide useful information for suicide prevention in LMICs by critically describing what has hitherto been the focus of research on poverty and suicide in the English literature in order to identify possible future directions for research. Unlike in our recently published systematic review summarising the findings of 37 papers reporting statistically significant associations between poverty and suicidal behaviour in LMICs (Iemmi *et al.*, 2016), our intention in this paper was to focus on methodological issues (such as measurement, study design, methods of statistical analysis and theoretical frameworks) and propose a research agenda for this field. We focused on poverty because this problem is endemic in LMICs and narrowed our search to monetary related measures of poverty (such as unemployment, wealth and indebtedness) in order to provide sufficient focus for the paper to be useful. We were interested in identifying studies that included poverty as either the independent or dependant variable. We included the full range of suicidal behaviour, namely suicidal ideation (i.e. thinking about death, wishing to be dead or planning one's death), non-fatal suicidal behaviours (i.e. taking steps towards ending one's life or deliberate self-harm) and completed suicides.

We systematically searched 11 databases; CINHAL Plus, EconLit, EMBASE, Global Health, HTA Database, IBSS, NHSEED, PsycINFO, MEDLINE, PAIS International, and Web of Science. A combination of key words for suicidal behaviour, poverty and LMICs were used. Searches were conducted for studies with abstract and full-text in English, published between January 2004 and April 2014. Additional searches were performed through snowballing and citation tracking of included studies. We included the full range of suicidal behaviour, but excluded studies focusing on assisted suicide, and studies that only examined exposures relating to violence, terrorism and war. We focused on monetary-related poverty indicators at the micro-economic (individual) and macro-economic (country) level. We excluded studies defining poverty through non-monetary indicators (e.g. education, health, type of housing and living conditions). We included LMICs identified using the World Bank Atlas method (World Bank, 2013).

We searched for a wide range of quantitative studies, including randomised controlled trials, quasi-randomised controlled trials, non-randomised controlled trials, before-and-after studies, interrupted-time series, cohort studies, case-control studies, cross-sectional studies, ecological studies, case report/case series, as well as economic evaluation and economic modelling studies. In the case of mixed method studies, we only included quantitative findings. Editorials, commentaries, book reviews, and review papers were excluded. In order to be included, studies had to report quantitative data on the relationship between poverty and suicide.

Two authors independently double screened title and abstract. Full-texts of all included studies were retrieved. Three authors independently double screened full-texts. Disagreements were discussed and a third author was consulted if needed.

Authors double-extracted data from all included studies to record the following: study characteristics; methodology, suicide dimensions; poverty dimensions; and relationship between suicide and poverty. Extracted data were summarised in table format and interpreted using narrative analysis.

## Results

A total of 3653 records were initially identified, of which duplicates (*n* = 1544) were excluded to yield 2109 records. These were screened by title and abstract, resulting in the retrieval and screening of 187 full-texts, of which 83 met the inclusion criteria. One additional article was identified through citation tracking and was included, giving a total of 84 included studies.

### Study characteristics

The characteristics of included studies by WHO region is provided in the accompanying online Supplementary Resource (Appendix 1). There has been a sharp increase in the number of English publications over the last decade, with numbers almost doubling after 2008. There was an uneven distribution of studies across regions of the world; studies tended to be concentrated in middle-income countries. The South-East Asia Region was the most researched region; 30% of studies were conducted in this region. Only 2% of studies were conducted in the Americas and 7% of studies drew data from multiple locations. There was an under-representation of low-income countries; 6% of studies were conducted in low income countries.

More than half the study designs were cross-sectional and the majority (60%) took place in community-based settings. Over half of the individuals evaluated across the studies were women, and less than one third (31%) were children under the age of 13. The majority of studies did not employ a theoretical framework to interpret data.

### Dimensions of poverty

The majority of the studies investigated individual level poverty dimensions, mainly unemployment (65%), economic status and wealth assets (39%), and economic/financial problems (27%). Table 1 shows which of the included studies reported on each of these individual level poverty dimensions. Studies that focused on individual level dimensions of poverty tended to examine the relationship between suicidal behaviour and current financial circumstances (e.g. current employment status or current level of debt); three studies gave attention to the longitudinal dimensions of poverty and suicidal behaviour. One study examined recent major financial crises (Manoranjitham *et al.* 2010). One study examined sudden economic bankruptcy, chronic financial problems, poverty in last 12 months and poverty since childhood (Gururaj *et al.* 2004) and one study examined family economic status in the previous year (Gong *et al.* 2011). But even these three studies failed to track the development of suicidal behaviour over time and could thus not draw conclusions about temporal relationships. Poverty was predominantly investigated as a proximal risk factor with inadequate attention given to the possible cumulative effects of poverty over time and how economic factors may act as a distal risk factor for suicidal behaviour, mediated by other more proximal factors such as psychological distress or interpersonal conflict.

**Table 1.** Individual level poverty measures, by poverty dimension (n = 72)

	Studies which utilised self-report measures	Studies which utilised reports by family, acquaintance or psychological autopsy	Studies which employed standardised questionnaires^a^	Studies which employed administrative records	Studies which employed official statistics
Relative poverty			Borges *et al.* (2010) (World Health Organisation Composite International Diagnostic Interview)	Grigoriev *et al.* (2013)	
Economic status and wealth assets	Dai *et al.* (2011), Gong *et al.* (2011), Joshi *et al.* (2010), Kinyanda *et al.* (2004), Ma *et al.* (2009), Manoranjitham *et al.* (2010), Mukhopadhyay *et al.* (2012), Qaisar *et al.* (2014), Saddichha *et al.* (2010), Thanh *et al.* (2006), Toprak *et al.* (2011), Toros *et al.* (2004), Wan *et al.* (2011), Zhang *et al.* (2006)	Feroz *et al.* (2012), Gedela (2008), Kaur *et al.* (2010), Kong & Zhang (2010), Mashreky *et al.* (2013), Mohanty *et al.* (2007), Sauvaget *et al.* (2009)	Pawan *et al.* (2012) (Kuppuswami Socioeconomic Status Scale)	Keyvanara *et al.* (2013), Polatöz *et al.* (2011)	
Unemployment	Ahmadi *et al.* (2009), Bansal & Barman (2011), Chowdhury *et al.* (2010), Ekramzadeh *et al.* (2012), Fleischmann *et al.* (2005), Ghaleiha *et al.* (2012), Keyvanara *et al.* (2013), Kinyanda *et al.* (2004), Manoranjitham *et al.* (2010), Nojomi *et al.* (2007), Nojomi *et al.* (2008), Ovuga *et al.* (2005), Qaisar *et al.* (2014), Ramim *et al.* (2013), Sabzghabaee *et al.* (2013), Sadr *et al.* (2013), Tahir *et al.* (2010), Thanh *et al.* (2006), Zaheer *et al.* (2009), Zhang & Zhou (2009)	Ahmadi *et al.* (2008), Fernando *et al.* (2010), Gururaj *et al.* (2004), Kale (2011*a*), Khan *et al.* (2008), Mashreky *et al.* (2013), Tahir *et al.* (2013)	Borges *et al.* (2010) (World Health Organisation Composite International Diagnostic Interview), Naidoo & Schlebusch (2013) (World health Organisation-validated questionnaire from SUPRE-MISS study)	Adinkrah (2011), Alimohammadi *et al.* (2013), Almasi *et al.* (2009), Aydin & Kartal (2010), Demirci *et al.* (2009), Drevinja *et al.* (2013), Lari *et al.* (2007), Nojomi *et al.* (2006), Singh (2009)	Aliverdinia & Pridemore (2009), Grigoriev *et al.* (2013), Manuel *et al.* (2008), Stevovic *et al.* (2011), Yur'yev *et al.* (2012)
Economic/financial problems	Du Toit *et al.* (2008), Hemmat *et al.* (2004), Hong *et al.* (2007), Khan *et al.* (2008), Kinyanda *et al.* (2004), Manoranjitham *et al.* (2010), Moosa *et al.* (2005), Nath *et al.* (2012), Xie *et al.* (2012), Zaheer *et al.* (2009)	Aydin & Kartal (2010), Gururaj *et al.* (2004), Kale (2011*b*), Lari *et al.* (2007), Lari *et al.* (2009), Mohanty (2005), Parkar *et al.* (2012), Stevovic *et al.* (2011)	Borges *et al.* (2010) (World Health Organisation Composite International Diagnostic Interview)		
Debt		Gedela (2008), Gururaj *et al.* (2004), Kale (2011*b*), Kaur *et al.* (2010), Mohanty (2005), Nagthan *et al.* (2011)			Kale (2011*a*)
Support from the welfare system					Aliverdinia & Pridemore (2009)

a Standardised measures are shown in brackets.

Comparatively few (*n* = 12) studies focused on macro-economic measures of poverty, namely economic crisis (2%), national income (11%), and composite measures of poverty (1%). Table 2 shows which of the included studies investigated each of these macro-economic dimensions of poverty. No studies investigated the impact of economic inequality on suicidal behaviour.

**Table 2.** Country level poverty measures, by poverty dimension (N = 12)

	Studies which utilised self-report measures	Studies which utilised reports by family, acquaintance or psychological autopsy	Studies which employed Standardised questionnaires^a^	Studies which employed administrative records	Studies which employed official statistics
Economic crisis		Kale (2011*a*, *b*)			
National income					Afroz *et al.* (2012), Altinanahtar & Halicioglu (2009), Bando *et al.* (2012), Blasco-Fontecilla *et al.* (2012), Botha (2012), Faria *et al.* (2006), Moniruzzaman & Andersson (2008*a*), Pandey & Kaur (2009), Zhang *et al.* (2010)
Composite poverty measure					Faria *et al.* (2006)

a Standardised measures are shown in brackets.

No studies reported on how suicidal behaviour may contribute to poverty; all the studies examined poverty as a potential independent variable and no studies considered the economic impact of suicidal behaviour (e.g. loss of family income due to injury, disability or death).

### Measures of poverty

Poverty was measured in a variety of ways across the included studies. Table 1 provides a summary of the measures of individual level dimensions of poverty; full details are provided in online Supplementary Appendix 2.

Fifteen studies reported on economic status and wealth assets, measured by self- or family-reports of individual or family monthly or annual income, value of family livestock assets, income generated from agriculture, and perceptions of family socio-economic and financial status. Thirteen studies reported on unemployment, measured by family self-report, official records (police reports), or official regional unemployment rates. Seven studies include self or family reported economic or financial adversity, including sudden economic bankruptcy, chronic financial problems, poverty in last 12 months, poverty since childhood, financial difficulties, recent major financial crisis, perceived level of stress due to economic circumstances, and financial burdens as a result of a medical condition. Three studies reported on debt, measured by family members' reports of outstanding debt per hectare of land owned, presence of large loans, and total amount of outstanding loan. The two studies which reported on relative poverty measured this concept as the proportion of family income relative to the national poverty line.

Macro-economic dimensions of poverty received substantially less attention than micro-economic measures. See Table 2 for a summary of which of the included studies reported on macro-economic level dimensions of poverty (full details are provided in online Supplementary Appendix 2). One study reported on support from the welfare system, which was measured as the percentage of the population receiving state welfare system support according to official statistics (Aliverdinia & Pridemore, 2009). This lack of attention to welfare systems probably reflects the general lack of welfare safety nets in LMICs. Two country-level measures of poverty were reported, national income (Moniruzzaman & Andersson, 2008*b*) and a composite measure of poverty (Faria *et al.* 2006). Changes in national income were measured by changes in official government statistics or World Bank reports of *per capita* income, purchasing power parity adjusted or real GDP *per capita*, and inflation rates. One study utilised the Human Development Index as a composite measure of poverty (Faria *et al.* 2006).

### Dimensions of suicidal behaviour

The majority of studies (*n* = 47) focused on non-fatal suicidal behaviours (i.e. suicidal ideation, intent, plan or attempt), compared with 37 studies which focused on suicide.

### Measures of suicidal behaviour

Details of the measures used to quantify suicides are summarised in Table 3; full details are provided in online Supplementary Appendix 3. Completed suicides were measured by the number of suicide deaths reflected in official records, death registers, police records, hospital records and the WHO mortality database.

**Table 3.** Measures used in studies which reported on fatal suicidal behaviour (i.e. suicide)

Studies which utilised self-report measures	Studies which utilised reports by family, acquaintance or psychological autopsy	Studies which employed Standardised questionnaires^a^	Studies which employed administrative records	Studies which employed official statistics
Eskandarieh *et al.* (2013)	Feroz *et al.* (2012)	Mashreky *et al.* (2013) (Household interview using National and Dhaka metropolitan survey)	Adinkrah (2011), Alimohammadi *et al.* (2013), Aliverdinia & Pridemore (2009), Almasi *et al.* (2009), Aydin & Kartal (2010), Bando *et al.* (2012), Demirci *et al.* (2009), Drevinja *et al.* (2013), Gedela (2008), Gururaj *et al.* (2004), Kale (2011*a*), Kaur *et al.* (2010), Khan *et al.* (2008), Lari *et al.* (2007), Manuel *et al.* (2008), Parkar *et al.* (2012), Singh (2009), Stevovic *et al.* (2011)	Afroz *et al.* (2012), Ahmadi *et al.* (2008), Altinanahtar & Halicioglu (2009), Blasco-Fontecilla *et al.* (2012), Botha (2012), Faria *et al.* (2006), Fernando *et al.* (2010), Grigoriev *et al.* (2013), Jena *et al.* (2009), Kong & Zhang (2010), Manoranjitham *et al.* (2010), Mohanty (2005), Mohanty *et al.* (2007), Moniruzzaman & Andersson (2008*a*), Nagthan *et al.* (2011), Pandey & Kaur (2009), Sauvaget *et al.* (2009), Tahir *et al.* (2013), Yur'yev *et al.* (2012), Yur'yev *et al.* (2012)

a Standardised measures are shown in brackets.

Details of the measures used to quantify non-fatal suicidal behaviour are provided in Table 4; full details are provided in online Supplementary Appendix 3. Suicidal ideation was measured by self-reports of thoughts of suicide, suicidal intention and suicide plans over different periods (1 week, 2 weeks, 6 months, 12 months or lifetime). Other forms of non-fatal suicidal behaviour (i.e. excluding suicidal ideation) were measured by self-reports of planned and unplanned suicide attempts or self-injuries over different periods of time (1 week, 2 weeks, 6 months, 12 months or lifetime) or from hospital records following self-injury.

**Table 4.** Measures used in studies which reported on non-fatal suicidal behaviour and suicidal ideations

Studies which utilised self-report measures	Studies which utilised reports by family, acquaintance or psychological autopsy	Studies which employed Standardised questionnaires^a^	Studies which employed administrative records	Studies which employed official statistics
Du Toit *et al.* (2008), Gong *et al.* (2011), Hong *et al.* (2007), Joshi *et al.* (2010), Ma *et al.* (2009), Mukhopadhyay *et al.* (2012), Nath *et al.* (2012), Qaisar *et al.* (2014), Ramim *et al.* (2013), Sabzghabaee *et al.* (2013), Toprak *et al.* (2011), Toros *et al.* (2004), Wan *et al.* (2011), Zhang & Zhou (2009)	Feroz *et al.* (2012)	Xie *et al.* (2012) (Beck Depression Inventory, Family APGAR and Trait Coping Style Questionnaire), Ovuga *et al.* (2005); Ekramzadeh *et al.* (2012) (Beck Scale for Suicide Ideation), Zhang & Zhou (2009) (Beck's Suicide Intent Scale), European Parasuicide Study, Fleischmann *et al.* (2005); Kinyanda *et al.* (2004) (Interview Schedule), Ekramzadeh *et al.* (2012) (Harmful Behaviour Scale), Kinyanda *et al.* (2011) (Mini International Neuropsychiatric Interview for children and adolescents), Dai *et al.* (2011) (National Comorbidity Survey), Zhang *et al.* (2006) (Semi-structured questionnaire including 8 questions selected from Beck's Suicidal Intent Scale), Polatöz *et al.* (2011) (Suicidal Behaviour Questionnaire), Polatöz *et al.* (2011) (Suicidal Ideation Scale), Polatöz *et al.* (2011) (Suicidal Intent Scale), Borges *et al.* (2010) (World Health Organisation Composite International Diagnostic Interview), Nojomi *et al.* (2007) (World Health Organisation SUPRE-MISS questionnaire), Thanh *et al.* (2006) (World Health Organisation SUPRE-MISS questionnaire)	Ahmadi *et al.* (2009), Bansal & Barman (2011), Chowdhury *et al.* (2010), Ghaleiha *et al.* (2012), Hemmat *et al.* (2004), Keyvanara *et al.* (2013), Lari *et al.* (2009), Manuel *et al.* (2008), Moosa *et al.* (2005), Naidoo & Schlebusch (2013), Nojomi *et al.* (2006), Nojomi *et al.* (2008), Pawan *et al.* (2012), Saddichha *et al.* (2010), Sadr *et al.* (2013), Tahir *et al.* (2010), Zaheer *et al.* (2009)	Tahir *et al.* (2013)

a Standardised measures are shown in brackets.

The following instruments were used to obtain self-reports of non-fatal suicidal behaviour: MINI International Neuropsychiatric Interview for children and adolescents; Suicidal Behaviour Questionnaire; Suicidal Intent Scale; Suicidal Ideation Scale; SUPRE-MISS community survey questionnaire; WHO Composite International Diagnostic Interview; Harmful Behaviour Scale; Beck Scale for Suicidal Ideation; and the Beck Depression Inventory.

### Statistical analysis

The statistical methods employed to analyse data in the included studies were, for the most part, unsophisticated and there was an absence of theory-driven analysis. The majority of the studies (*n* = 47) employed descriptive statistics making it impossible to draw firm inferences about the nature of the relationship between poverty and suicidal behaviour. A total of 44.6% (*n* = 37) of the studies employed bivariate or multivariate statistical analysis, thus establishing statistically meaningful associations between poverty and suicidal behaviour.

## Discussion

Our findings suggest there is a small but growing English literature describing the relationship between poverty and suicidal behaviour in LMICs. There are significant knowledge gaps in this field, thus highlighting where future research might be focused. More robust evidence is needed in this area, with future studies giving attention to measurement issues, focusing on a wider range of poverty dimensions, utilising more sophisticated statistical methods, focusing on individual experiences of poverty rather than aggregate level measures, taking account of temporal factors and employing theory-driven research.

The studies in this field are not evenly distributed across geographic and economic regions; there is a dearth of research from sub-Saharan Africa, from the poorest countries of the world, and from countries with the highest rates of suicide (e.g. Guyana). The uneven distribution of studies may in part reflect our decision to only include studies published in English; 14 studies were excluded because they were not in English. This calls attention to the need for researchers with the requisite language skills to conduct systematic mappings of the literature published in other languages; doing so is necessary to complete the picture of what is known about poverty and suicidal behaviour in LMICs.

Studies are needed in those countries, which have hitherto been neglected to expand the knowledge base in this field. This is particularly apt in light of evidence that cultural context is important in the aetiology of suicide, suggesting that findings in one country cannot simply be extrapolated to another setting (Chang *et al.* 2009; Colucci & Lester, 2012; WHO, 2014). The lack of research from these regions may well be a function of resource limitations but it may also partly reflect stigma and the criminalisation of suicidal behaviour in some LMICs; as is the case in Guyana, Uganda and Ghana. It may be important to explore the extent to which stigma and attitudes towards suicide hinder research of this topic and to undertake advocacy work to generate evidence within LMICs where suicide is highly stigmatised or criminalised.

Wide variations in the way poverty and suicidal behaviour were measured make it difficult to synthesise findings. Some dimensions of poverty, for example, unemployment, are measured relatively consistently. Other dimensions of poverty, most notably relative poverty and economic status, are inconsistently measured. Similarly, comparatively few studies make use of standardised measurement instruments to assess suicidal behaviour. Where instruments are used we find that there are nine different psychometric instruments used reporting suicidal behaviour over a variety of different time periods. Further evidence of the lack of a standardised nomenclature is apparent in the variety of ways in which non-fatal suicidal behaviour is operationalised in the included studies; some studies focus on self-harm, others employ the concept of suicide attempt, while others focus on very specific kinds of self-harm, such as self-inflicted burns. It would be helpful if future studies employed standardised and widely used instruments to measure suicidal behaviour and poverty in order to facilitate the integration and comparison of research findings. The need for more robust measures and consistencies across studies has been highlighted in recent publications for both suicide (Jordans *et al.* 2014) and poverty (Cooper *et al.* 2012).

There are measurement problems associated with trying to quantify suicidal ideation, which is a fluctuating phenomenon that varies according to the dimensions such as frequency, intensity and duration (Posner *et al*. 2014; Wang *et al*. 2014). The studies included in this systematic mapping tend to treat suicidal ideation as a stable and unitary construct. Future work in this area will need to consider how to make meaningful comparisons between fluctuations in suicidal ideation and enduring economic factors such as poverty. In this context, there may be lessons to be learned from the work being done on mobile and real-time assessments, and ecological momentary assessments of predictors of non-fatal suicidal behaviour; see for example, the work of Armey (2012) and Ben-Zeev *et al.* (2012).

The lack of statistical sophistication in this field of study is noteworthy and may, at least in part, reflect limitations of data available about suicidal behaviour and poverty in many LMICs. Even studies that made use of bivariate or multivariate statistical analysis simply investigated associations between poverty and suicidal behaviour, without attempting to theorise about the relationship between these variables. The majority of studies did not control for potential confounding variables such as gender, divorce rates, civil status, alcohol consumption and level of education, all of which are known to influence rates of suicide. No studies took account of how psychological factors (such as the experience of shame or feelings of powerlessness) might act as mediating factors in the relationship between poverty and suicidal behaviour. This may in part be indicative of the fact that the measurement of concepts such as shame is currently much less well-developed than, for example, categories such as employment or socio-economic status. In this context, it is significant to note that the ways in which people experience (and report) poverty are strongly influenced by factors, including culture, social class, status and gender. It would be helpful if future studies took into account the effect of possible mediating psychological and cultural factors.

There is a need for future studies in this area to focus on the subjective experiences of individuals in LMICs who have engaged in suicidal behaviour. Individual level and qualitative studies may draw more nuanced understandings of the relationship between poverty and suicidal behaviour than those which result from aggregate level ecological studies. In this respect, it may be helpful for future work in this area to include psycho-social autopsy studies of completed suicide and qualitative studies of medically serious suicide attempts which consider experiences of poverty.

The lack of any effort to understand temporal relationships between poverty and suicidal behaviour is problematic. Most of the studies report on the presence of poverty and suicidal behaviour without attempting to place these within a meaningful timeframe or to document the onset of these phenomena. For example, studies that report an association between family income and suicidal behaviour make no effort to understand if affected individuals have lived in poverty all their lives or have recently fallen into poverty. Likewise, it is not clear if the suicidal behaviour reported predates or follows the occurrence of a poverty-related dimension such as unemployment. This may in part reflect the difficulties collecting longitudinal data in LMICs. Studies also fail to take account of contextual factors such as the level of income inequality in communities and the extent to which this might mediate the association between poverty and suicidal behaviour. These are significant shortcomings, especially in the light of research, which suggests that the effects on suicide of economic shocks may be delayed; higher rates of suicide were most pronounced after the end of the 1990s economic crisis in Sweden by individuals who experienced long-term unemployment (Garcy & Vagero, 2013). There are opportunities given the rapid development being experienced in some LMICs to conduct longitudinal analyses on the impacts not only of economic downturns, but also rapid economic growth, and how any resulting widening of inequalities and/or changing social structures impact on suicidal behaviours. In this respect, it may be helpful to build on the work of Blasco-Fontecilla *et al.* (2012) addressing the impact of economic cycles on suicide.

It is significant that most studies in this field are conceptualised within the paradigm of risk-factor models, reporting on associations between variables without attempting to theorise about why there would be a relationship between specific dimensions of poverty and suicidal behaviour. Furthermore, none of the studies took account of the potential mediating effects of the psychological experience of poverty, which may help account for links between poverty and suicidal behaviour. Future studies might seek to explore these psychological phenomena and their role in links between poverty and suicidal behaviour. Suggestions for how this might be achieved are provided in Table 5. Some work has already been undertaken in this area by economists in HICs (e.g. the work of Suzuki (2008) on economic uncertainty and suicide in Australia and the work of Korhonen *et al.* (2014) on hardship and suicide in Finland); this kind of analysis with appropriate cultural modifications could be extended into LMICs.

**Table 5.** Examples of variables that might be captured in future epidemiological studies, which seek to understand the relationship between suicidal behaviour and poverty

Theoretical models of suicidal behaviour	Psychological constructs which may mediate the relationship between poverty and SIB	Variables
Integrated Motivational Volitional model (O'Connor, 2011)	Feelings of entrapment	Subjective experience of feeling trapped by poverty
Shneidman's (1998) model	Suicidal behaviour as a perceived solution to an insoluble problem	Subjective perception of how suicide is a solution to the real life problems that result from living under conditions of poverty
Interpersonal-psychological theory (Joiner, 2005)	Thwarted belonging.	Subjective experience of thwarted belonging and perceived burdensomeness as a result of living under conditions of poverty.
	Perceived burdensomeness.	Subjective experience of feeling hopeless about any possibility for a change in economic circumstances
	Hopelessness	

Our findings have important implications for future research in this area. Analyses need to be consistently disaggregated by both gender and age, so as to be better able to identify associations between poverty and suicidal behaviour across the life course, where feasible analyses also need to take into account factors, such as the availability of different forms of social safety nets and the availability of social capital resources, including support from families, community organisations and religious/faith-based groups.

It may be possible to perform *post hoc* secondary analysis using multivariate analysis of data in studies, which only made use of descriptive statistics, provided that their datasets are still available. This may be an important and cost effective line of enquiry given the high number of studies that employ descriptive statistics and the relative paucity of studies using more sophisticated statistical analysis and modelling.

It would be helpful if future studies were able to provide insight into the mechanism by which poverty precipitates suicidal behaviour in some individuals and not in others living under conditions of economic hardship. To answer these questions future studies need to be theory driven and will need to integrate qualitative research methods to explore the subjective lived-experience of poverty among individuals who engage in suicidal behaviour. Box 1 provides a summary of the suggestions for future research in this field.

Box 1. Recommendations for future research on the relationship between suicidal behaviour and poverty in LMIC.

Relevant contextual variables, such as availability of social security, need to be clearly identified.

Pay attention to measurement issues and clearly operationalise poverty and suicidal behaviour in standardised ways that facilitate subsequent meta-analysis of data.

Statistical analysis should move beyond descriptive statistics to allow for relationships between variables to be appropriately explored.

Epidemiological data should be consistently broken down by gender and age.

Include longitudinal studies that allow for the exploration of the temporal relationship between poverty variables and suicidal behaviour.

Explore differences between individuals living in poverty who engage in suicidal behaviour and those who do not, including documenting factors that promote resilience.

Where appropriate utilise theoretical approaches to the design and analysis of epidemiological studies.

Include measures of appropriate psychological constructs (such as feeling of entrapment and perceived burdensomeness) along with demographic and economic data in epidemiological studies on poverty and suicide.

Where appropriate include qualitative studies that explore subjective psychological and lived experience of individuals living in poverty who engage in suicidal behaviour.

## Limitations

A limitation of this paper is the exclusion of studies not published in English; this paper thus presents at best a partial picture and runs the risk of perpetuating the impression that the only knowledge we have of global mental health issues is that which is published in English journals. A further limitation is our decision to narrow our searches to monetary-related measures of poverty in order to give the paper more focus. Poverty is a multi-dimensional construct and there are a number of non-monetary-related dimensions of poverty (e.g. living circumstances, access to health care and access to education), which potentially have a bearing on suicidal behaviour.

## Conclusion

This study provides a systematic mapping of the research published in English exploring links between poverty and suicidal behaviour in LMICs. Our data show that while there is a growing body of research in this area, there are a number of significant gaps in the literature and more sophisticated theory-driven studies are needed, which move beyond simply describing associations.

## Supplementary material

For supplementary material accompanying this paper visit http://dx.doi.org/10.1017/gmh.2016.27. click here to view supplementary material
