# Supplementary material for: Poverty and suicide research in low- and middle-income countries: systematic mapping of literature published in English and a proposed research agenda
Source: Glob Ment Health (Camb). 2016 Dec 13;3:e32. doi: 10.1017/gmh.2016.27 (PMC5454768; doi:10.1017/gmh.2016.27)
Supplement: Supplementary file 1 [file S2054425116000273sup001.docx]

**Appendix 1**

Characteristics of included studies, by WHO region (N=84)

| **First author** | **Setting** | **Study population** | **Study design (sample size)** | **Poverty dimension** | **Suicide dimension** | **Analysis** |
| --- | --- | --- | --- | --- | --- | --- |
| **AFRO** |  |  |  |  |  |  |
| Adinkrah et al.., 2011 | Ghana  Rural | Children, adolescents, adults and older adults (n/a age)  5% female | Case report/case series (n=287) | Unemployment | Fatal suicide | Descriptive |
| Botha et al., 2012 | South Africa  Community based (rural and urban) | Adolescents, adults and older adults (15-96 years old)  n/a gender | Economic modelling (n=1659) | National income | Fatal suicide | Bivariate |
|  |  |  |  | National income | Fatal suicide | Multivariate |
| du Toit et al., 2008 | South Africa  Hospital based | Children, adolescents, adults and older adults (7-87 years old)  69% female | Retrospective cohort study (n=259) | Economic/ financial problems | Non-fatal suicide | Descriptive |
| Jena et al., 2009 | South Africa  Community based (urban) | Adolescents and adults (13+ years old)  3% female | Case report/case series (n=46) | Unemployment | Fatal suicide | Descriptive |
| Kinyanda et al., 2004 | Uganda  Hospital based (urban) | Adolescents, adults and older adults (13+ years old)  37% female | Case-control study (n=400) | Economic/ financial problems | Non-fatal suicide | Descriptive |
|  |  |  |  | Unemployment | Non-fatal suicide | Descriptive |
| Kinyanda et al., 2011 | Uganda  Community based (rural) | Children, adolescents and adults (0-19 years old)  53% female | Cross-sectional study (n=1492) | Economic status and wealth assets | Non-fatal suicide | Bivariate |
| Moosa et al., 2005 | South Africa  Hospital based (urban) | Adolescents, adults and older adults (13+ years old)  60% female | Cross-sectional study (n=43) | Economic/ financial problems | Non-fatal suicide | Descriptive |
| Naidoo et al., 2013 | South Africa  Hospital based | Adolescents and adults (13-64 years old)  75% female | Cross-sectional study (n=688) | Unemployment | Non-fatal suicide | Descriptive |
| Ovuga et al., 2005 | Uganda  Community based (rural) | Adults and older adults (19+ years old)  33% female | Cross-sectional study (n=939) | Unemployment | Non-fatal suicide | Bivariate |
| **AMRO** |  |  |  |  |  |  |
| Bando et al., 2012 | Brazil  Community based (rural and urban) | Adolescents, adults and older adults (15+ years old)  20% female | Interrupted-time series (n=98904) | National income | Fatal suicide | Bivariate |
| Faria et al., 2006 | Brazil  Community based (peri-urban) | Adolescents, adults and older adults (13+ years old)  20% female | Ecological study (n=4766) | Composite poverty measure | Fatal suicide | Multivariate |
|  |  |  |  | National income | Fatal suicide | Multivariate |
| **EMRO** |  |  |  |  |  |  |
| Ahmadi et al., 2008 | Iran  Community based (rural and urban) | Children, adolescents, adults and older adults (n/a age)  71% female | Controlled ecological study (n=4267) | Unemployment | Fatal suicide | Descriptive |
| Ahmadi et al., 2009 | Iran  Hospital based (rural and urban) | n/a age  87% female | Case-control study (n=60) | Unemployment | Non-fatal suicide | Multivariate |
| Alimohammadi et al., 2013 | Iran  Hospital based | Adolescents, adults and older adults (13-74 years old)  20% female | Cross-sectional study (n=79) | Unemployment | Fatal suicide | Descriptive |
| Aliverdinia et al., 2009 | Iran  Community based (urban) | n/a age  100% female | Ecological study (n=100) | Support from the welfare system | Fatal suicide | Multivariate |
|  |  |  |  | Unemployment | Fatal suicide | Multivariate |
| Ekramzadeh et al., 2012 | Iran  Hospital based | Adults and older adults (19+ years old), medically ill  39% female | Cross-sectional study (n=650) | Unemployment | Non-fatal suicide | Bivariate |
|  |  |  |  | Unemployment | Non-fatal suicide | Multivariate |
|  |  |  |  | Unemployment | Non-fatal suicide | Bivariate |
|  |  |  |  | Unemployment | Non-fatal suicide | Multivariate |
| Eskandarieh et al., 2012 | Iran  Community based (rural and urban) | Children, adolescents, adults and older adults (n/a age)  64% female | Cross-sectional study (n=892) | Unemployment | Fatal suicide | Descriptive |
|  |  |  |  | Unemployment | Non-fatal suicide | Descriptive |
| Ghaleiha et al., 2012 | Iran  Hospital based (rural and urban) | Children, adolescents, adults and older adults (n/a age)  53% female | Cross-sectional study (n=1566) | Unemployment | Non-fatal suicide | Descriptive |
| Hemmat et al., 2004 | Iran  Hospital based | Adolescents, adults and older adults (13+ years old)  99% female | Cohort study (n=412) | Economic/ financial problems | Non-fatal suicide | Descriptive |
| Keyvanara et al., 2013 | Iran  Hospital based | Adolescents, adults and older adults (13+ years old)  74% female | Cross-sectional study (n=179) | Economic status and wealth assets | Non-fatal suicide | Descriptive |
| Keyvanara et al., 2013 | Iran  Hospital based | Adolescents, adults and older adults (13+ years old)  74% female | Cross-sectional study (n=179) | Unemployment | Non-fatal suicide | Descriptive |
| Khan et al., 2008 | Pakistan  Community based | n/a age  17% female | Case-control study (n=200) | Economic/ financial problems | Fatal suicide | Bivariate |
|  |  |  |  | Unemployment | Fatal suicide | Bivariate |
| Lari et al., 2007 | Iran  Hospital based (urban) | Adolescents and adults (13-64 years old)  79% female | Cross-sectional study (n=89) | Economic/ financial problems | Fatal suicide | Descriptive |
|  |  |  |  | Unemployment | Fatal suicide | Descriptive |
|  |  |  |  | Economic/ financial problems | Non-fatal suicide | Descriptive |
|  |  |  |  | Unemployment | Non-fatal suicide | Descriptive |
| Lari et al., 2009 | Iran  Hospital based (urban) | Children, adolescents, adults and older adults (n/a age)  72% female | Cohort study (n=125) | Economic/ financial problems | Fatal suicide | Descriptive |
|  |  |  |  | Economic/ financial problems | Non-fatal suicide | Descriptive |
| Nojomi et al., 2006 | Iran  Hospital based (urban) | n/a age  63% female | Cross-sectional study (n=632) | Unemployment | Non-fatal suicide | Descriptive |
| Nojomi et al., 2007 | Iran  Community based (urban) | Children, adolescents, adults and older adults (n/a age)  65% female | Cross-sectional study (n=2300) | Unemployment | Non-fatal suicide | Bivariate |
|  |  |  |  | Unemployment | Non-fatal suicide | Multivariate |
| Nojomi et al., 2008 | Iran  Hospital based (urban) | Children, adolescents, adults and older adults (n/a age)  63% female | Cross sectional study (n=632) | Unemployment | Non-fatal suicide | Descriptive |
| Qaisar et al., 2014 | Pakistan  Hospital based (urban) | Adolescents, adults and older adults (13+ years old)  60% female | Cross-sectional study (n=100) | Economic status and wealth assets | Non-fatal suicide | Descriptive |
|  |  |  |  | Unemployment | Non-fatal suicide | Descriptive |
| Ramim et al., 2013 | Iran  Hospital based | Adolescents and adults (13-64 years old), married  100% female | Cohort study (n=35) | Unemployment | Non-fatal suicide | Descriptive |
| Sabzghabaee et al., 2013 | Iran  Hospital based (rural and urban) | Adolescents and adults (13-64 years old)  60% female | Cohort study (n=400) | Unemployment | Non-fatal suicide | Descriptive |
| Sadr et al., 2013 | Iran  Hospital based (urban) | Adolescents and adults (13-64 years old), multiple suicide attempters  57% female | Cross-sectional study (n=400) | Unemployment | Non-fatal suicide | Descriptive |
| Tahir et al., 2010 | Pakistan  Hospital based | n/a age  62% female | Case report/case series (n=154) | Unemployment | Non-fatal suicide | Descriptive |
| Tahir et al., 2013 | Pakistan  Community based (rural and urban) | Children, adolescents and adults (0-64 years old)  22% female | Cohort study (n=108) | Unemployment | Fatal suicide | Descriptive |
|  |  |  |  | Unemployment | Non-fatal suicide | Descriptive |
| **EUR** |  |  |  |  |  |  |
| Grigoriev et al., 2013 | Belarus  Community based (rural and urban) | Adolescents and adults (13-64 years old)  n/a gender | Ecological study (n=n/a) | Relative poverty | Fatal suicide | Multivariate |
|  |  |  |  | Unemployment | Fatal suicide | Multivariate |
| Almasi et al., 2009 | Hungary  Urban and rural | Adults (36-55 years old)  68% female | Case-control study (n=388) | Unemployment | Fatal suicide | Bivariate |
| Altinanahtar et al., 2009 | Turkey  Community based (rural and urban) | n/a age  n/a gender | Economic modelling (n=n/a) | National income | Fatal suicide | Multivariate |
| Aydin et al., 2010 | Turkey  Community based | Adolescents, adults and older adults (11-88 years old)  41% female | Cross-sectional study (n=169) | Economic/ financial problems | Fatal suicide | Descriptive |
|  |  |  |  | Unemployment | Fatal suicide | Descriptive |
| Demirci et al., 2009 | Turkey  Community based | Adults and older adults (19+ years old)  38% female | Case report/case series (n=16) | Unemployment | Fatal suicide | Descriptive |
| Drevinja et al., 2013 | Kosovo  Community based | n/a age  n/a gender | Case report/case series (n=270) | Unemployment | Fatal suicide | Descriptive |
| Polatöz et al., 2011 | Turkey  Community based | Adolescents and adults (13-64 years old)  55% female | Cross-sectional study (n=1117) | Economic status and wealth assets | Non-fatal suicide | Bivariate |
| Stevovic et al., 2011 | Serbia  Community based | Adolescents, adults and older adults (13+ years old)  27% female | Cross-sectional study (n=303) | Economic/ financial problems | Fatal suicide | Descriptive |
|  |  |  |  | Unemployment | Fatal suicide | Descriptive |
| Toprak et al., 2011 | Turkey  Community based (rural and urban) | Adolescents and adults (13-64 years old) , students  54% female | Cross-sectional study (n=636) | Economic status and wealth assets | Non-fatal suicide | Bivariate |
|  |  |  |  | Economic status and wealth assets | Non-fatal suicide | Bivariate |
|  |  |  |  | Economic status and wealth assets | Non-fatal suicide | Multivariate |
| Toros et al., 2004 | Turkey  School based (rural and urban) | Children, adolescents and adults (10-20 years old)  n/a gender | Cross-sectional study (n=4143) | Economic status and wealth assets | Non-fatal suicide | Bivariate |
| **SEARO** |  |  |  |  |  |  |
| Bansal et al., 2011 | India  Hospital based (rural and urban) | Adolescents, adults and older adults (13+ years old)  58% female | Cross-sectional study (n=100) | Unemployment | Non-fatal suicide | Descriptive |
| Chowdhury et al., 2010 | India  Clinic based (rural and urban) | Children, adolescents, adults and older adults (n/a age)  62% female | Case report/case series (n=1614) | Unemployment | Non-fatal suicide | Descriptive |
| Fernando et al., 2010 | Sri Lanka  Hospital based | Children, adolescents, adults and older adults (n/a age)  38% female | Cross-sectional study (n=151) | Unemployment | Fatal suicide | Descriptive |
| Feroz et al., 2012 | Bangladesh  Community based (rural) | Adolescents, adults and older adults (13+ years old)  74% female | Cross-sectional study (n=3551 households; n=12422 individuals) | Economic status and wealth assets | Fatal suicide | Descriptive |
|  |  |  |  | Economic status and wealth assets | Fatal suicide | Descriptive |
|  |  |  |  | Economic status and wealth assets | Fatal suicide | Descriptive |
|  |  |  |  | Economic status and wealth assets | Non-fatal suicide | Descriptive |
|  |  |  |  | Economic status and wealth assets | Non-fatal suicide | Descriptive |
|  |  |  |  | Economic status and wealth assets | Non-fatal suicide | Descriptive |
| Gedela et al., 2008 | India  Community based (rural) | n/a age, farmers  n/a gender | Cross-sectional study (n=74) | Debt | Fatal suicide | Multivariate |
|  |  |  |  | Economic status and wealth assets | Fatal suicide | Multivariate |
|  |  |  |  | Economic status and wealth assets | Fatal suicide | Multivariate |
| Gururaj et al., 2004 | India  Community based (urban) | Children, adolescents, adults and older adults (n/a age)  33% female | Case-control study (n=538) | Debt | Fatal suicide | Bivariate |
|  |  |  |  | Economic/ financial problems | Fatal suicide | Bivariate |
|  |  |  |  | Unemployment | Fatal suicide | Bivariate |
|  |  |  |  | Unemployment | Fatal suicide | Multivariate |
| Joshi et al., 2010 | India  Hospital based (rural and urban) | Children, adolescents and adults (0-64 years old)  33% female | Cross-sectional study (n=350) | Economic status and wealth assets | Non-fatal suicide | Descriptive |
| Kale et al., 2011a | India  Community based (rural) | n/a age, farmers  n/a gender | Cross-sectional study (n=200) | Debt | Fatal suicide | Descriptive |
|  |  |  |  | Economic crisis | Fatal suicide | Descriptive |
|  |  |  |  | Unemployment | Fatal suicide | Descriptive |
| Kale et al., 2011b | India  Community based (rural) | n/a age, farmers  n/a gender | Case-control study (n=80) | Debt | Fatal suicide | Descriptive |
|  |  |  |  | Economic crisis | Fatal suicide | Descriptive |
|  |  |  |  | Economic/ financial problems | Fatal suicide | Descriptive |
| Kaur et al., 2010 | India  Community based (rural) | n/a age, farmers  n/a gender | Case-control study (n=120 families) | Debt | Fatal suicide | Bivariate |
|  |  |  |  | Economic status and wealth assets | Fatal suicide | Bivariate |
| Manoranjitham et al., 2010 | India  Community based (rural) | Children, adolescents, adults and older adults (n/a age)  41% female | Case-control study (n=200) | Economic status and wealth assets | Fatal suicide | Bivariate |
|  |  |  |  | Economic/ financial problems | Fatal suicide | Bivariate |
|  |  |  |  | Unemployment | Fatal suicide | Bivariate |
| Manuel et al., 2008 | Sri Lanka  Hospital based (rural) | Adolescents and adults (13-64 years old)  n/a gender | Ecological study (n=189) | Unemployment | Fatal suicide | Multivariate |
|  |  |  |  | Unemployment | Non-fatal suicide | Multivariate |
| Mashreky et al., 2013 | Bangladesh  Community based (rural and urban) | Children, adolescents, adults and older adults (n/a age)  55% female | Cross-sectional study (n=171366 households) | Economic status and wealth assets | Fatal suicide | Descriptive |
|  |  |  |  | Unemployment | Fatal suicide | Descriptive |
| Mohanty et al., 2005 | India  Community based (rural) | n/a age, farmers  n/a gender | Interrupted-time series (n=66) | Debt | Fatal suicide | Descriptive |
|  |  |  |  | Economic/ financial problems | Fatal suicide | Descriptive |
| Mohanty et al., 2007 | India  Hospital and community based (rural and urban) | Children, adolescents, adults and older adults (n/a age)  n/a gender | Retrospective cohort study (n=588) | Economic status and wealth assets | Fatal suicide | Descriptive |
| Mukhopadhyay et al., 2012 | India  Community based (rural and urban) | Adolescents (13-18 years old)  30% female | Cross-sectional study (n=2068) | Economic status and wealth assets | Non-fatal suicide | Multivariate |
| Nagthan et al., 2011 | India  Community based (rural) | Adults and older adults (19+ years old), farmers  3.34% female | Case-control study (n=60) | Debt | Fatal suicide | Descriptive |
| Nath et al., 2012 | India  Community based (urban) | Adolescents and adults (13-64 years old), students  61% female | Cross-sectional study (n=1817) | Economic/ financial problems | Non-fatal suicide | Multivariate |
| Pandey et al., 2009 | India  Community based (rural and urban) | n/a age  n/a gender | Economic modelling (n=n/a) | National income | Fatal suicide | Multivariate |
| Parkar et al., 2012 | India  Community based (urban) | Children, adolescents and adults (0-64 years old)  56% female | Case report/case series (n=50) | Economic/ financial problems | Fatal suicide | Descriptive |
| Pawan et al., 2012 | India  Hospital based (rural and urban) | Children, adolescents and adults (0-64 years old)  43% female | Cross-sectional study (n=89) | Economic status and wealth assets | Non-fatal suicide | Descriptive |
| Saddichha et al., 2010 | India  Community based (rural and urban) | Children, adolescents, adults and older adults (n/a age)  44% female | Cross-sectional study (n=40541) | Economic status and wealth assets | Non-fatal suicide | Descriptive |
| Sauvaget et al., 2009 | India  Community based (rural) | Adults and older adults (35+ years old)  62% female | Cohort study (n=131720) | Economic status and wealth assets | Fatal suicide | Bivariate |
|  |  |  |  | Economic status and wealth assets | Fatal suicide | Multivariate |
| Singh et al., 2009 | India  Community based (rural and urban) | Children, adolescents, adults and older adults (n/a age)  40% female | Interrupted-time series (n=1298) | Unemployment | Fatal suicide | Descriptive |
| Zaheer et al., 2009 | India  Hospital based (rural and urban) | Children, adolescents, adults and older adults (n/a age)  40% female | Cross-sectional study (n=104) | Economic/ financial problems | Non-fatal suicide | Descriptive |
|  |  |  |  | Unemployment | Non-fatal suicide | Descriptive |
| **WPRO** |  |  |  |  |  |  |
| Dai et al., 2011 | China  Community based (rural) | Adolescents and adults (16-34 years old)  53% female | Cross-sectional study (n=1654) | Economic status and wealth assets | Non-fatal suicide | Multivariate |
|  |  |  |  | Economic status and wealth assets | Non-fatal suicide | Multivariate |
| Gong et al., 2011 | China  Community based (rural) | Adolescents, adults and older adults (13+ years old)  61% female | Cross-sectional study (n=3821) | Economic status and wealth assets | Non-fatal suicide | Bivariate |
|  |  |  |  | Economic status and wealth assets | Non-fatal suicide | Multivariate |
| Hong et al., 2007 | China  Community based (rural) | Children, adolescents, adults and older adults (n/a age), sex workers  100% female | Cross-sectional study (n=454) | Economic/ financial problems | Non-fatal suicide | Multivariate |
| Kong & Zhang, 2010 | China  Community based (rural) | Children and adults (13-64 years old)  47% female | Case-control study (n=740) | Economic status and wealth assets | Fatal suicide | Bivariate |
| Ma et al., 2009 | China  Community based (rural and urban) | Adolescents, adults and older adults (13+ years old)  54% female | Cross-sectional study (n=5926) | Economic status and wealth assets | Non-fatal suicide | Bivariate |
|  |  |  |  | Economic status and wealth assets | Non-fatal suicide | Multivariate |
| Thanh et al., 2006 | Vietnam  Community based (urban) | Adolescents, adults and older adults (13+ years old)  52% female | Cross-sectional study (n=2280) | Economic status and wealth assets | Non-fatal suicide | Bivariate |
|  |  |  |  | Economic status and wealth assets | Non-fatal suicide | Multivariate |
|  |  |  |  | Unemployment | Non-fatal suicide | Bivariate |
| Wan et al., 2011 | China  School based (urban) | Adolescents (13-18 years old)  51% female | Cross-sectional study (n=17622) | Economic status and wealth assets | Non-fatal suicide | Bivariate |
| Xie et al., 2012 | China  Hospital based | Adolescents, adults and older adults (13+ years old), patients with systemic lupus erythematosus  94% female | Cross-sectional study (n=285) | Economic/ financial problems | Non-fatal suicide | Multivariate |
|  |  |  |  | Economic/ financial problems | Non-fatal suicide | Bivariate |
| Zhang et al., 2006 | China  Hospital based (urban) | n/a age  46% female | Case-control study (n=166) | Economic status and wealth assets | Non-fatal suicide | Bivariate |
| Zhang et al., 2009 | China  Community based (rural) | n/a age  46% female | Case-control study (n=132) | Unemployment | Non-fatal suicide | Descriptive |
| Zhang et al., 2010 | China  Community based (rural and urban) | n/a age  n/a gender | Interrupted-time series (n=n/a) | National income | Fatal suicide | Multivariate |
| **Multiple** |  |  |  |  |  |  |
| Afroz et al., 2012 | 3 countries  Thailand  [*Japan, South Korea]  Community based (rural and urban) | Adults (15-44 years old)  n/a gender | Ecological study (n=n/a) | National income | Fatal suicide | Descriptive |
| Blasco-Fontecilla et al., 2012 | 56 countries  AMR B (Argentina, Brazil, Chile, Colombia, Costa Rica, El Salvador, Mexico, Panama, St. Lucia, Trinidad and Tobago and Venezuela) AMR D (Ecuador and Guatemala) SEAR D (India) WPR B (China and South Korea) AFR D (Mauritius) [*AMR A (Canada and the USA) EUR A (Austria, Belgium, Croatia, Czech Republic, Denmark, Finland, France, Germany, Greece, Iceland, Ireland, Israel, Italy, Luxembourg, Malta, Netherlands, Norway, Portugal, Slovenia, Spain, Sweden, Switzerland and the UK) EUR B (Bulgaria, Georgia, Macedonia, Poland, Romania and the Slovak Republic) EUR C (Estonia, Hungary, Latvia and Lithuania); WPR A (Australia, New Zealand, Japan and Singapore)]  Community based (rural and urban) | n/a age  n/a gender | Interrupted-time series (n=n/a) | National income | Fatal suicide | Bivariate |
| Borges et al., 2010 | 21 countries  Brazil, Bulgaria, China, Colombia, India, Lebanon, Mexico, Nigeria, Romania, South Africa, Ukraine;  [*Belgium, France, Germany, Israel, Italy, Japan, Netherlands, New Zealand, Spain, United States]  Community based (rural and urban) | Adults (n/a age)  n/a gender | Cross-sectional study (n=108705) | Unemployment | Non-fatal suicide | Bivariate |
|  |  |  |  | Economic/ financial problems | Non-fatal suicide | Bivariate |
|  |  |  |  | Economic/ financial problems | Non-fatal suicide | Bivariate |
|  |  |  |  | Relative poverty | Non-fatal suicide | Bivariate |
|  |  |  |  | Relative poverty | Non-fatal suicide | Bivariate |
|  |  |  |  | Unemployment | Non-fatal suicide | Bivariate |
| Fleischmann et al., 2005 | 8 countries  Brazil, China, Estonia, India, Iran, South Africa, Sri Lanka, Vietnam  Hospital based (rural and urban) | Children, adolescents, adults and older adults (n/a age)  60.5% female | Cross-sectional study (n=4314) | Unemployment | Non-fatal suicide | Descriptive |
| Moniruzzaman et al., 2008 | 66 countries  Low-income countries: Armenia, Azerbaijan, Georgia, Kyrgyzstan, Rep Moldova, Ukraine, Uzbekistan Middle-income countries: Albania, Argentina, Belarus, Brazil, Bulgaria, Chile, Colombia, Costa Rica, Croatia, Cuba, Czech Rep, Ecuador, Egypt, El Salvador, Estonia, Guatemala, Hungary, Kazakhstan, Latvia, Lithuania, Macedonia, Mauritius, Mexico, Panama, Paraguay, Philippines, Poland, Romania, Rush Fed, Slovakia, Slovenia, Thailand, Uruguay, Venezuela  [*High-income countries: Australia, Austria, China (HK), Denmark, Finland, Germany, Greece, Ireland, Israel, Italy, Japan, Kuwait, Netherlands, New Zealand, Norway, Portugal, Puerto Rico, Rep Korea, Singapore, Spain, Sweden, Switzerland, UK, USA]  Community based | Children, adolescents, adults and older adults (n/a age)  n/a gender | Cross-sectional study (n=66 countries) | National income | Fatal suicide | Descriptive |
| Yur'yev et al., 2012 | 26 countries  Bulgaria Hungary Romania Ukraine  [*Belgium, Croatia, the Czech Republic, Denmark, Estonia, Finland, France, Germany, Greece, Israel, the Latvia, Netherlands, Norway, Poland, Portugal, the Russian Federation, Slovakia, Slovenia, Spain, Sweden, Switzerland and the UK]  Community based | n/a age  n/a gender | Cross-sectional study (n=26 countries) | Unemployment | Fatal suicide | Descriptive |

Note: WHO regions: region of the Americas (AMRO), African region (AFRO), Eastern Mediterranean region (EMRO), European region (EURO), South-East Asia region (SEARO), and the Western Pacific region (WPRO). n/a Not available. ^a^Study quality: high (++), acceptable (+), low (-).

**Appendix 2**

Poverty indicators and measures, by poverty dimension (N=84)

| Poverty dimension | Study | Setting | Poverty indicator | Poverty measure |
| --- | --- | --- | --- | --- |
| Individual level | | | | |
| Relative poverty | Borges et al., 2010 | 21 countries | Family income defined into four categories based on the ratio of income to number of family members relative to the official poverty line | self-reported as part of WHO Composite International Diagnostic Interview |
|  | Grigoriev, Doblhammer-Reiter, & Shkolnikov, 2013 | Belarus | Proportion of people with income below the poverty line | Income and Expenditure of Households Sample Survey |
| Economic status and wealth assets | Dai et al., 2011 | China | Perceived financial status | self-reported |
|  | Feroz et al., 2012 | Bangladesh | Wealth measured by family land ownership classified as landless, <2 acres, 2 or more acres | as reported by heads of households |
|  |  |  | Socio-economic status measured as lower, lower-middle, middle or upper class on the basis of a composite score of: earning capacity, housing status, possession of essential and luxury goods in the family |  |
|  |  |  | Monthly combined family income |  |
|  | Gedela, 2008 | India | Value of livestock | as reported by family |
|  |  |  | Value of agricultural produce |  |
|  | Gong, Zhang, Wang, & Liang, 2011 | China | Family economic status in the previous year, defined as good, fair or poor | self-reported |
|  | Joshi, Joshi, Nigam, Joshi, & Prakash, 2010 | India | Socio-economic status classified as low, middle or high | self-reported |
|  | Kaur, Dhaliwal, & Singh, 2010 | India | Income from dairy farming, income from non-farming sources, and income from crops | as reported by family |
|  | Keyvanara, Mousavi, & Karami, 2013 | Iran | Socio-economic status | composite measure of economic status determined by: patient’s income, education, accommodation, occupation; his/her spouse’ job; house and land ownership as well as car type and model; variety of consumption and purchase of equipment for home (such as clothes and other necessities); personal consumptions; and the person’s life style and family life style (such as travel, food, sport and using outdoor foods) |
|  | Kinyanda, Hjelmeland, & Musisi, 2004 | Uganda | Family’s total income per month (in Uganda shillings) | self-reported |
|  | Kong & Zhang, 2010 | China | Annual family income | as collected via psychological autopsy |
|  | Ma et al., 2009 | China | Monthly income | self-reported |
|  | Manoranjitham et al., 2010 | India | Monthly family income less than 2400 Indian Rupees | self-reported |
|  | Mashreky, Rahman, & Rahman, 2013 | Bangladesh | Monthly family income | as reported by family |
|  | Mohanty, Sahu, Mohanty, & Patnaik, 2007 | India | Socio-economic status categorized into three categories, depending on the income per family per year (i.e. up to Rupees 50,000 = lower, Rupees 50,001 to 1,50,000 = middle, and more than Rupees 1,50,000 = upper) | as reported by victim's acquaintances |
|  | Mukhopadhyay, Mukhopadhyay, Sinhababu, & Biswas, 2012 | India | Perceived economic status (SES) was categorized as high (very well off/living comfortably) or low (just getting by/almost poor/poor) | self-reported |
|  | Pawan, Mohan, Kalita, & Nitin, 2012 | India | Socio-economic status classified as Upper, Upper-Middle, Lower-Middle, Upper-Lower, Lower | as measured by Kuppuswami scale of socioeconomic classification |
|  | Polatöz, Kuğu, Doğan, & Akyüz, 2011 | Turkey | Level of family income, classified as high, middle and low | Socio-demographic information form |
|  | Qaisar et al., 2014 | Pakistan | Socioeconomic status | self-reported |
|  | Saddichha, Vibha, Saxena, & Methuku, 2010 | India | Socio-economic status classified as above or below the poverty line on the basis of family monthly income | self-report |
|  | Sauvaget et al., 2009 | India | Monthly household income | as reported by family |
|  | Thanh, Trung, Jiang, Leenaars, & Wasserman, 2006 | Vietnam | Socio-economic status classified as high or low on the basis of mean monthly family income | self-reported |
|  | Toprak, Cetin, Guven, Can, & Demircan, 2011 | Turkey | Level of income, classified as low or high | self-reported |
|  | Toros, Bilgin, Sasmaz, Bugdayci, & Camdeviren, 2004 | Turkey | Family monthly income | self-reported |
|  | Wan, Hu, Hao, Sun, & Tao, 2011 | China | Perceived family economic status | self-reported |
|  | Zhang, Jia, Jiang, & Sun, 2006 | China | Perception of socioeconomic status | self-reported |
| Unemployment | Adinkrah, 2011 | Ghana | Employment status and type of employment | police records |
|  | Ahmadi, Mohammadi, Stavrinos, Almasi, & Schwebel, 2008 | Iran | Employment status | as reported by family members |
|  | Ahmadi et al., 2009 | Iran | Employment status | self-reported |
|  | Alimohammadi, Mehrpisheh, & Memarian, 2013 | Iran | Employment status | mortuary records |
|  | Aliverdinia & Pridemore, 2009 | Iran | Unemployment rate among working aged females | official statistics |
|  | Almasi et al., 2009 | Hungary | Employment status | police records |
|  | Aydin & Kartal, 2010 | Turkey | Employment status | official judicial records |
|  | Bansal & Barman, 2011 | India | Employment status | self-reported |
|  | Borges et al., 2010 | 21 countries | Employment status | self-reported as part of WHO Composite International Diagnostic Interview |
|  | Chowdhury et al., 2010 | India | Reasons for engaging in deliberate self-harm | self-reported |
|  | Demirci, Dogan, Erkol, & Deniz, 2009 | Turkey | Employment status | as recorded in mortuary records |
|  | Drevinja, Berisha, Serreqi, Statovci, & Haxhibeqiri, 2013 | Kosovo | Employment status | as recorded in hospital records |
|  | Ekramzadeh et al., 2012 | Iran | Employment status | self-reported |
|  | Fernando, Hewagama, Priyangika, Range, & Karunaratne, 2010 | Sri Lanka | Employment status | as reported by close friend or family |
|  | Fleischmann et al., 2005 | 8 countries | Employment status | self-reported |
|  | Ghaleiha, Afzali, Bazyar, Khorsand, & Torabian, 2012 | Iran | Employment status | self-reported |
|  | Grigoriev, Doblhammer-Reiter, & Shkolnikov, 2013 | Belarus | Unemployment rate | official statistics |
|  | Gururaj, Isaac, Subbakrishna, & Ranjani, 2004 | India | Employment status | as collected via psychological autopsy with family members |
|  | Jena, Mountany, & Muller, 2009 | South Africa | Employment status | official records |
|  | Kale, 2011a | India | Employment status | as collected via psychological autopsy with family members |
|  | Keyvanara, Mousavi, & Karami, 2013 | Iran | Employment status | self-reported |
|  | Khan, Mahmud, Karim, Zaman, & Prince, 2008 | Pakistan | Employment status | as collected via psychological autopsy with friends or family members |
|  | Kinyanda, Hjelmeland, & Musisi, 2004 | Uganda | Employment status | self-reported |
|  | Lari, Joghataei, Adli, Zadeh, & Alaghehbandan, 2007 | Iran | Employment status | hospital records |
|  | Manoranjitham et al., 2010 | India | Employment status | self-reported |
|  | Manuel et al., 2008 | Sri Lanka | Unemployment rate | official statistics |
|  | Mashreky, Rahman, & Rahman, 2013 | Bangladesh | Employment status | as reported by family |
|  | Naidoo & Schlebusch, 2013 | South Africa | Employment status | self-reported, as measured by a WHO-validated questionnaire |
|  | Nojomi et al., 2006 | Iran | Employment status | as recorded in hospital records |
|  | Nojomi et al., 2007 | Iran | Employment status | self-reported |
|  | Nojomi et al., 2008 | Iran | Employment status | self-reported |
|  | Ovuga, Boardman, & Wassermann, 2005 | Uganda | Employment status | self-reported |
|  | Qaisar et al., 2014 | Pakistan | Employment status | self-reported |
|  | Ramim, Mobayen, Shoar, Naderan, & Shoar, 2013 | Iran | Employment status | self-reported |
|  | Sabzghabaee et al., 2013 | Iran | Employment status | self-reported |
|  | Sadr et al., 2013 | Iran | Employment status | self-reported |
|  | Singh, 2009 | India | Employment status | as recorded in hospital records |
|  | Stevovic, Jasovic-Gasic, Vukovic, Pekovic, & Terzic, 2011 | Serbia | Employment status | official records |
|  | Tahir, Memon, Kumar, & Ali, 2010 | Pakistan | Employment status | self-reported |
|  | Tahir et al., 2013 | Pakistan | Employment status | as reported by family |
|  | Thanh, Trung, Jiang, Leenaars, & Wasserman, 2006 | Vietnam | Employment status | self-reported |
|  | Yur'yev, Vaernik, Vaernik, Sisask, & Leppik, 2012 | 26 countries | National employment rates | official statistics |
|  | Zaheer, Aslam, Vibhanshu, Vibhor, & Khan, 2009 | India | Employment status | self-reported |
|  | Zhang & Zhou, 2009 | China | Employment status | self-reported |
| Economic/financial problems | Aydin & Kartal, 2010 | Turkey | Economic problems | as reported by parents of suicide victim |
|  | Borges et al., 2010 | 21 countries | Economic adversity | self-reported as part of WHO Composite International Diagnostic Interview |
|  | du Toit et al., 2008 | South Africa | Reasons for attempted suicide | self-reported |
|  | Gururaj, Isaac, Subbakrishna, & Ranjani, 2004 | India | Sudden economic bankruptcy; chronic financial problems; poverty in last 12 months and poverty since childhood | as collected via psychological autopsy with family members |
|  | Hemmat et al., 2004 | Iran | Reasons given for self-immolation | self-reported |
|  | Hong, Li, Fang, & Zhao, 2007 | China | Financial concerns | self-reported |
|  | Kale, 2011b | India | Gap between farmer’s income and liabilities | as reported by family |
|  | Khan, Mahmud, Karim, Zaman, & Prince, 2008 | Pakistan | Financial difficulties | as collected via psychological autopsy with friends or family members |
|  | Kinyanda, Hjelmeland, & Musisi, 2004 | Uganda | Financial difficulties | self-reported |
|  | Lari, Joghataei, Adli, Zadeh, & Alaghehbandan, 2007 | Iran | Reason given for self-burning | as collected via psychological autopsy with family members |
|  | Lari, Alaghehbandan, Panjeshahin, & Joghataei, 2009 | Iran | Reason given for self-burning | as collected via psychological autopsy with family members |
|  | Manoranjitham et al., 2010 | India | History of recent major financial crisis | self-reported |
|  | Mohanty, 2005 | India | Reason given for the farmer’s suicide | as collected via psychological autopsy with family members |
|  | Moosa, Jeenah, & Vorster, 2005 | South Africa | Financial difficulties | self-reported |
|  | Nath, Paris, Thombs, & Kirmayer, 2012 | India | Perceived level of stress due to economic circumstances | self-reported |
|  | Parkar, Nagarsekar, & Weiss, 2012 | India | Reports of financial problems/debt | as collected via sociocultural autopsy with living relatives |
|  | Stevovic, Jasovic-Gasic, Vukovic, Pekovic, & Terzic, 2011 | Serbia | Reasons given for suicide | as collected via psychological autopsy with friends or family members |
|  | Xie et al., 2012 | China | Financial burden of having lupus (categorised as no, low, moderate, and heavy burden) | self-reported |
|  | Zaheer, Aslam, Vibhanshu, Vibhor, & Khan, 2009 | India | Financial difficulties | self-reported |
| Debt | Gedela, 2008 | India | Outstanding debt per hectare | as reported by family |
|  | Gururaj, Isaac, Subbakrishna, & Ranjani, 2004 | India | Presence of large loan | as collected via psychological autopsy with family members |
|  | Kale, 2011a | India | Value of debt | official records |
|  | Kale, 2011b | India | Value of farmers debt | as reported by family |
|  | Kaur, Dhaliwal, & Singh, 2010 | India | Total loan outstanding | as reported by family |
|  | Mohanty, 2005 | India | Reason given for the farmer’s suicide (crop losses and indebtedness, farmers unable to recover costs of cultivation) | as collected via psychological autopsy with family members |
|  | Nagthan, Rajendra, Kunnal, Basvaraja, & Basavaraj, 2011 | India | Financial debt | as reported by family |
| Support from the welfare system | Aliverdinia & Pridemore, 2009 | Iran | Percentage of the population receiving support from the Iranian welfare system | official statistics |
| Country level | | | | |
| Economic crisis | Kale, 2011a | India | Financial circumstances | as collected via psychological autopsy with family members |
|  | Kale, 2011b | India | Annual income | as reported by family |
| National income | Afroz, Moniruzzaman, Stark Ekman, & Andersson, 2012 | 3 countries | GNP per capita | official statistics |
|  | Altinanahtar & Halicioglu, 2009 | Turkey | Per capita real income | official statistics |
|  | Bando, Brunoni, Bensenor, & Lotufo, 2012 | Brazil | GDP per capita | official statistics |
|  | Blasco-Fontecilla et al., 2012 | 56 countries | PPP-adjusted GDP per capita (PPP = purchasing power parity) | as obtained from the World Bank official statistics |
|  | Botha, 2012 | South Africa | Inflation rate in each region as a proxy for economic performance | official statistics |
|  | Faria, Victora, Meneghel, De Carvalho, & Falk, 2006 | Brazil | GDP per capita | official statistics |
|  | Moniruzzaman & Andersson, 2008 | 66 countries | Mean Gross National Income per capita | official statistics |
|  | Pandey & Kaur, 2009 | India | GDP per capita | official statistics |
|  | Zhang et al., 2010 | China | Per capita GDP adjusted for inflation | official statistics |
| Composite poverty measure | Faria, Victora, Meneghel, De Carvalho, & Falk, 2006 | Brazil | HDI-income (HDI = Human Development Index) | official statistics |

**Appendix 3**

SIB indicators and measures, by SIB dimension (fatal/non-fatal SIB) (N=84)

| Study | Setting | SIB indicator | SIB measure |
| --- | --- | --- | --- |
| Fatal SIB | | | |
| Adinkrah, 2011 | Ghana | Number of fatal suicides | police records |
| Afroz, Moniruzzaman, Stark Ekman, & Andersson, 2012 | 3 countries | National rate of fatal suicide | WHO mortality database |
| Ahmadi, Mohammadi, Stavrinos, Almasi, & Schwebel, 2008 | Iran | Rates of self-immolation for each province in Iran | official records |
| Alimohammadi, Mehrpisheh, & Memarian, 2013 | Iran | Number of fatal suicides | mortuary records |
| Aliverdinia & Pridemore, 2009 | Iran | Number of fatal suicides | Women’s Participation Centre Records |
| Almasi et al., 2009 | Hungary | Number of fatal suicides | police records |
| Altinanahtar & Halicioglu, 2009 | Turkey | Number of fatal suicides | official records |
| Aydin & Kartal, 2010 | Turkey | Number of fatal suicides | official judicial records |
| Bando, Brunoni, Bensenor, & Lotufo, 2012 | Brazil | Number of fatal suicides | death registry databases |
| Blasco-Fontecilla et al., 2012 | 56 countries | Suicide rate | WHO mortality database |
| Botha, 2012 | South Africa | Number of fatal suicides | official death notification |
| Demirci, Dogan, Erkol, & Deniz, 2009 | Turkey | Number of fatal suicides | mortuary records |
| Drevinja, Berisha, Serreqi, Statovci, & Haxhibeqiri, 2013 | Kosovo | Number of fatal suicides | hospital records |
| Eskandarieh, Hajebi, Saberi-Zafarghandi, Vares-Vazirian, & Asadi, 2012 | Iran | Number of fatal suicides | self-reported |
| Faria, Victora, Meneghel, De Carvalho, & Falk, 2006 | Brazil | Number of fatal suicides | official records of cause of death |
| Fernando, Hewagama, Priyangika, Range, & Karunaratne, 2010 | Sri Lanka | Number of fatal suicides | official judicial records of cause of death |
| Feroz et al., 2012 | Bangladesh | Number of fatal suicides | reported by heads of households |
| Gedela, 2008 | India | Number of fatal suicides | local legal death registers |
| Grigoriev, Doblhammer-Reiter, & Shkolnikov, 2013 | Belarus | Number of fatal suicides | National Committee of Statistics of Belarus |
| Gururaj, Isaac, Subbakrishna, & Ranjani, 2004 | India | Number of fatal suicides | police records |
| Jena, Mountany, & Muller, 2009 | South Africa | Number of fatal suicides | official Medico-Legal Laboratory records |
| Kale, 2011a | India | Number of fatal suicides | death registers |
| Kaur, Dhaliwal, & Singh, 2010 | India | Number of fatal suicides | police records |
| Khan, Mahmud, Karim, Zaman, & Prince, 2008 | Pakistan | Number of fatal suicides | police records |
| Kong & Zhang, 2010 | China | Number of fatal suicides | official records |
| Lari, Joghataei, Adli, Zadeh, & Alaghehbandan, 2007 | Iran | Number of fatal suicides by self-inflicted burn | hospital records |
| Manoranjitham et al., 2010 | India | Number of fatal suicides | official suicide surveillance system |
| Manuel et al., 2008 | Sri Lanka | Number of fatal suicides | hospital records |
| Mashreky, Rahman, & Rahman, 2013 | Bangladesh | Number of fatal suicides | household interview using National and Dhaka metropolitan surveys |
| Mohanty, 2005 | India | Number of fatal suicides over 3 year period in Amravati and Yavatmal Districts | official death records |
| Mohanty, Sahu, Mohanty, & Patnaik, 2007 | India | Number of fatal suicides | official coroner records |
| Moniruzzaman & Andersson, 2008 | 66 countries | Number of fatal suicides | WHO mortality database |
| Nagthan, Rajendra, Kunnal, Basvaraja, & Basavaraj, 2011 | India | Number of fatal suicides | official records of Joint Director of Agriculture and Deputy Commissioner Offices |
| Pandey & Kaur, 2009 | India | National rate of fatal suicide | official records |
| Parkar, Nagarsekar, & Weiss, 2012 | India | Number of fatal suicides | police reports |
| Sauvaget et al., 2009 | India | Number of fatal suicides | official death register |
| Singh, 2009 | India | Number of fatal suicides | hospital records |
| Stevovic, Jasovic-Gasic, Vukovic, Pekovic, & Terzic, 2011 | Serbia | Number of fatal suicides | police records |
| Tahir, Memon, Kumar, & Ali, 2013 | Pakistan | Number of fatal suicides | official records of emergency services |
| Yur'yev, Vaernik, Vaernik, Sisask, & Leppik, 2012 | 26 countries | Number of fatal suicides | WHO European mortality database |
| Zhang et al., 2010 | China | National rates of fatal suicides | official records |
| Non-fatal SIB | | | |
| Ahmadi et al., 2009 | Iran | Hospital admission following intentional self-burning | hospital records |
| Bansal & Barman, 2011 | India | Number of hospital admissions for deliberate self-harm | hospital records |
| Borges et al., 2010 | 21 countries | 12 month planned and unplanned suicide attempt, suicide plan and suicidal ideation | self-reported as part of WHO Composite International Diagnostic Interview |
| Chowdhury et al., 2010 | India | Number of cases of self-harm | clinic records |
| Dai et al., 2011 | China | Suicide attempt | self-report as part of National Comorbidity Survey |
| Dai et al., 2011 | China | Suicidal ideation, serious ideation, suicide plan | self-report as part of National Comorbidity Survey |
| du Toit et al., 2008 | South Africa | Number of hospital admission for suicide attempt | self-reported |
| Ekramzadeh et al., 2012 | Iran | Non-fatal suicidal behaviours | self-reported in response to the Harmful Behaviour Scale; self-reported in response to Beck Scale for Suicidal Ideation Scale; self-reported |
| Feroz et al., 2012 | Bangladesh | Number of suicide attempts | reported by heads of households |
| Fleischmann et al., 2005 | 8 countries | Number of suicide attempts | European Parasuicide Study Interview Schedule |
| Ghaleiha, Afzali, Bazyar, Khorsand, & Torabian, 2012 | Iran | Number of hospital admissions for suicide attempts | hospital records |
| Gong, Zhang, Wang, & Liang, 2011 | China | Six-months prevalence of suicidal thoughts, suicidal attempts or self-injury | self-reported |
| Hemmat et al., 2004 | Iran | Number of suicide attempts by self-immolation | hospital records |
| Hong, Li, Fang, & Zhao, 2007 | China | Six-month prevalence of suicidal ideation or suicidal attempt among sex workers | self-reported |
| Joshi, Joshi, Nigam, Joshi, & Prakash, 2010 | India | Number of hospital admissions for suicide attempt | self-reported |
| Keyvanara, Mousavi, & Karami, 2013 | Iran | Hospital admissions for self-harm | hospital record |
| Kinyanda, Hjelmeland, & Musisi, 2004 | Uganda | Life-time prevalence of suicide attempt | European Parasuicide Study Interview Schedule |
| Kinyanda, Kizza, Levin, Ndyanabangi, & Abbo, 2011 | Uganda | Suicidal ideation, self-harm and suicide attempt | Mini International Neuropsychiatric Interview (MINI) for children and adolescents to measure adolescent suicidality |
| Lari, Alaghehbandan, Panjeshahin, & Joghataei, 2009 | Iran | Hospital admission for suicide attempt by self-inflicted burn | hospital record |
| Ma et al., 2009 | China | Life-time prevalence of suicidal ideation and plans, and suicidal attempts | self-reported |
| Manuel et al., 2008 | Sri Lanka | Hospital admission for suicide attempt | hospital records |
| Moosa, Jeenah, & Vorster, 2005 | South Africa | Hospital admission for suicide attempt | hospital records |
| Mukhopadhyay, Mukhopadhyay, Sinhababu, & Biswas, 2012 | India | 12-month prevalence of suicide ideation and attempt | self-reported |
| Naidoo & Schlebusch, 2013 | South Africa | Number of admissions to hospital following a suicide attempt | hospital records |
| Nath, Paris, Thombs, & Kirmayer, 2012 | India | Suicidal ideation or suicidal attempt | self-reported |
| Nojomi et al., 2006 | Iran | Hospital admission for suicide attempt | hospital records |
| Nojomi et al., 2007 | Iran | Suicide ideation, plans, attempts | WHO SUPRE-MISS questionnaire with specific questions on suicide and related factors |
| Nojomi et al., 2008 | Iran | Hospital admission for suicide attempt | hospital records |
| Ovuga, Boardman, & Wassermann, 2005 | Uganda | Life-time and one-week prevalence of suicidal ideation | Beck Scale for Suicide Ideation |
| Pawan, Mohan, Kalita, & Nitin, 2012 | India | Hospital treatment for self-harm | hospital records |
| Polatöz, Kuğu, Doğan, & Akyüz, 2011 | Turkey | Suicidal ideation and intent | Suicidal Behaviour Questionnaire, Suicidal Intent Scale and Suicidal Ideation Scale |
| Qaisar et al., 2014 | Pakistan | History of deliberate self-poisoning | self-reported |
| Ramim, Mobayen, Shoar, Naderan, & Shoar, 2013 | Iran | Attempted suicide by self-burning | self-reported |
| Sabzghabaee et al., 2013 | Iran | Hospital admission for attempted suicide by self-poisoning | self-reported |
| Saddichha, Vibha, Saxena, & Methuku, 2010 | India | Hospital admission for suicide attempt | hospital records |
| Sadr et al., 2013 | Iran | Hospital admissions following a suicide attempt | hospital records |
| Tahir, Memon, Kumar, & Ali, 2010 | Pakistan | Hospital admission for self-inflicted burn | hospital records |
| Tahir et al., 2013 | Pakistan | Number of suicide attempts | official records of emergency services |
| Thanh, Trung, Jiang, Leenaars, & Wasserman, 2006 | Vietnam | Lifetime and 12-month prevalence of prevalence suicidal thoughts | self-reported in response to SUPRE-MISS community survey questionnaire |
| Toprak, Cetin, Guven, Can, & Demircan, 2011 | Turkey | Life time prevalence of self-harm, suicidal ideation, suicidal attempt | self-reported |
| Toros, Bilgin, Sasmaz, Bugdayci, & Camdeviren, 2004 | Turkey | Suicide attempt | self-reported |
| Wan, Hu, Hao, Sun, & Tao, 2011 | China | 12-month prevalence of self-harm | self-reported |
| Xie et al., 2012 | China | Life-time and 12-month prevalence of suicidal ideation | Beck Depression Inventory, Family APGAR and Trait Coping Style Questionnaire |
| Zaheer, Aslam, Vibhanshu, Vibhor, & Khan, 2009 | India | Hospital admission for self-poisoning | hospital records |
| Zhang, Jia, Jiang, & Sun, 2006 | China | Suicide attempt | Semi-structured questionnaire including 8 questions selected from Beck's Suicidal Intent Scale |
| Zhang & Zhou, 2009 | China | Previous suicide attempts and suicidal ideation / intention | self-report; Beck’s Suicide Intent Scale |

**References**

Adinkrah, M. (2011). Epidemiologic characteristics of suicidal behavior in contemporary ghana. *Crisis: The Journal of Crisis Intervention & Suicide Prevension, 32*, 31-36.

Afroz, B., Moniruzzaman, S., Stark Ekman, D., & Andersson, R. (2012). The impact of economic crisis on injury mortality: The case of the 'Asian crisis'. *Public Health, 126*, 836-838.

Ahmadi, A., Mohammadi, R., Schwebel, D. C., Khazaie, H., Yeganeh, N., & Almasi, A. (2009). Demographic risk factors of self-immolation: a case-control study. *Burns, 35*, 580-586.

Ahmadi, A., Mohammadi, R., Stavrinos, D., Almasi, A., & Schwebel, D. C. (2008). Self-immolation in Iran. *Journal of Burn Care & Research, 29*, 451-460.

Alimohammadi, A. M., Mehrpisheh, S., & Memarian, A. (2013). Epidemiology of cases of suicide due to hanging who referred to forensic center of Shahriar in 2011. *International Journal of Medical Toxicology and Forensic Medicine, 3*, 121-125.

Aliverdinia, A., & Pridemore, W. A. (2009). Women's fatalistic suicide in Iran: a partial test of Durkheim in an Islamic republic. *Violence Against Women, 15*, 307-320.

Almasi, K., Belso, N., Kapur, N., Webb, R., Cooper, J., Hadley, S., et al. (2009). Risk factors for suicide in Hungary: a case-control study. *BMC Psychiatry, 9*, 45.

Altinanahtar, A., & Halicioglu, F. (2009). A dynamic econometric model of suicides in Turkey. *Journal of Socio-Economics, 38*, 903-907.

Aydin, B., & Kartal, M. (2010). Suicide cases in a province (Samsun) of Black Sea region of Turkey between 1999-2003 years. *Turkiye Klinikleri Journal of Medical Sciences, 30*, 1067-1072.

Bando, D. H., Brunoni, A. R., Bensenor, I. M., & Lotufo, P. A. (2012). Suicide rates and income in Sao Paulo and Brazil: A temporal and spatial epidemiologic analysis from 1996 to 2008. *BMC Psychiatry, 12*.

Bansal, P. D., & Barman, R. (2011). Psychiatric morbidity and the socio-demographic determinants of deliberate self harm. *Journal of Clinical and Diagnostic Research, 5*, 601-604.

Blasco-Fontecilla, H., Perez-Rodriguez, M. M., Garcia-Nieto, R., Fernandez-Navarro, P., Galfalvy, H., De Leon, J., et al. (2012). Worldwide impact of economic cycles on suicide trends over 3 decades: Differences according to level of development. A mixed effect model study. *BMJ Open, 2*.

Borges, G., Nock, M. K., Abad, J. M. H., Hwang, I., Sampson, N. A., Alonso, J., et al. (2010). Twelve-month prevalence of and risk factors for suicide attempts in the world health organization world mental health surveys. *Journal of Clinical Psychiatry, 71*, 1617-1628.

Botha, F. (2012). The economics of suicide in South Africa. *South African Journal of Economics, 80*, 526-552.

Chowdhury, A. N., Banerjee, S., Brahma, A., Das, S., Sarker, P., Biswas, M. K., et al. (2010). A prospective study of suicidal behaviour in Sundarban delta, West Bengal, India. *National Medical Journal of India, 23*, 201-205.

Dai, J., Chiu, H. F. K., Conner, K. R., Chan, S. S. M., Hou, Z. J., Yu, X., et al. (2011). Suicidal ideation and attempts among rural Chinese aged 16-34 years - Socio-demographic correlates in the context of a transforming China. *Journal of Affective Disorders, 130*, 438-446.

Demirci, S., Dogan, K. H., Erkol, Z., & Deniz, I. (2009). A series of complex suicide. *American Journal of Forensic Medicine & Pathology, 30*, 152-154.

Drevinja, F., Berisha, B., Serreqi, V., Statovci, S., & Haxhibeqiri, S. (2013). Suicides in kosovo in five year period 2008-2012, an overview for possible social motives. *European Psychiatry, 28*.

du Toit, E. H., Kruger, J. M., Swiegers, S. M., van der Merwe, M., Calilz, F. J. W., Philane, L., et al. (2008). The profile analysis of attempted-suicide patients referred to Pelonomi Hospital for psychological evaluation and treatment from 1 May 2005 to 30 April 2006. *South African Journal of Psychiatry, 14*, 20-26.

Ekramzadeh, S., Javadpour, A., Draper, B., Mani, A., Withall, A., & Sahraian, A. (2012). Prevalence and correlates of suicidal thought and self-destructive behavior among an elderly hospital population in Iran. *International Psychogeriatrics, 24*, 1402-1408.

Eskandarieh, S., Hajebi, A., Saberi-Zafarghandi, M. B., Vares-Vazirian, M., & Asadi, A. (2013). Demographic risk factors of suicide in Savojbolagh city of Tehran Province: 2007-2009. *Studies on Ethno Medicine, 7*, 143-148.

Faria, N. M. X., Victora, C. G., Meneghel, S. N., De Carvalho, L. A., & Falk, J. W. (2006). Suicide rates in the State of Rio Grande do Sul, Brazil: Association with socioeconomic, cultural, and agricultural factors. [Portuguese] Taxas de suicidio no Estado do Rio Grande do Sul, Brasil: Associacao com fatores socio-economicos, culturais e agrarios. *Cadernos de Saude Publica, 22*, 2611-2621.

Fernando, R., Hewagama, M., Priyangika, W. D., Range, S., & Karunaratne, S. (2010). Study of suicides reported to the Coroner in Colombo, Sri Lanka. *Medicine, Science, and the Law, 50*, 25-28.

Feroz, A. H. M., Islam, S. M. N., Selim, R., Rahman, A. K. M. M., Jawaharlal, S., Mogni, M., et al. (2012). A community survey on the prevalence of suicidal attempts and deaths in a selected rural area of Bangladesh. *Journal of Medicine, 13*, 3-9.

Fleischmann, A., Bertolote, J. M., Leo, D. d., Botega, N., Phillips, M., Sisask, M., et al. (2005). Characteristics of attempted suicides seen in emergency-care settings of general hospitals in eight low- and middle-income countries. *Psychological Medicine, 35*, 1467-1474.

Gedela, S. P. R. (2008). Factors responsible for agrarian crisis in Andhra Pradesh (a logistic regression analysis). *World Applied Sciences Journal, 4*, 707-713.

Ghaleiha, A., Afzali, S., Bazyar, M., Khorsand, F., & Torabian, S. (2012). Characteristics of hospitalized patients following suicide attempt in Hamadan District, Iran. *Oman Medical Journal, 27*, 304-309.

Gong, Y. H., Zhang, L., Wang, Z. Q., & Liang, Y. (2011). Pathway analysis of risk factors for severe suicidal ideation: A survey in rural China. *Canadian Journal of Public Health-Revue Canadienne De Sante Publique, 102*, 472-475.

Grigoriev, P., Doblhammer-Reiter, G., & Shkolnikov, V. (2013). Trends, patterns, and determinants of regional mortality in Belarus, 1990-2007. *Population Studies, 67*, 61-81.

Gururaj, G., Isaac, M. K., Subbakrishna, D. K., & Ranjani, R. (2004). Risk factors for completed suicides: a case-control study from Bangalore, India. *Injury control and safety promotion, 11*, 183-191.

Hemmat, M., Abasad, G., Jafary, G. A., Gila, A., Nahid, A., Bahram, K., et al. (2004). Women victims of self-inflicted burns in Tabriz, Iran. *Burns, 30*, 217-220.

Hong, Y., Li, X., Fang, X., & Zhao, R. (2007). Correlates of suicidal ideation and attempt among female sex workers in China. *Health Care for Women International, 28*, 490-505.

Jena, S., Mountany, L., & Muller, A. (2009). A demographic study of homicide-suicide in the Pretoria region over a 5 year period. *Journal of Forensic & Legal Medicine, 16*, 261-265.

Joshi, S. C., Joshi, A., Nigam, P., Joshi, G., & Prakash, C. (2010). Pattern of poisoning cases admitted at a tertiary care centre in the Kumaon region of Uttarakhand. *Indian Journal of Forensic Medicine and Toxicology, 4*, 4-5.

Kale, N. M. (2011). Productivity, annual income and indebtedness position: a comparative study of farmers who committed suicides with others. *Karnataka Journal of Agricultural Sciences, 24*, 343-346.

Kale, N. M. (2011). Socio-psycho risk factors associated with suicidal farmers: application of step down analysis. *Karnataka Journal of Agricultural Sciences, 24*, 700-703.

Kaur, P., Dhaliwal, H. S., & Singh, S. Y. (2010). Farmers' suicides in Punjab: a discriminant analysis approach. *The Indian Journal of Economics, 91*.

Keyvanara, M., Mousavi, S. G., & Karami, Z. (2013). Social class status and suicide characteristics: a survey among patients who attempted suicide in Isfahan. *Materia Socio Medica, 25*, 56-59.

Khan, M. M., Mahmud, S., Karim, M. S., Zaman, M., & Prince, M. (2008). Case-control study of suicide in Karachi, Pakistan. *British Journal of Psychiatry, 193*, 402-405.

Kinyanda, E., Hjelmeland, H., & Musisi, S. (2004). Deliberate self-harm as seen in Kampala, Uganda - a case-control study. *Social psychiatry and psychiatric epidemiology, 39*, 318-325.

Kinyanda, E., Kizza, R., Levin, J., Ndyanabangi, S., & Abbo, C. (2011). Adolescent suicidality as seen in rural Northeastern Uganda: Prevalence and risk factors. *Crisis, 32*, 43-51.

Kong, Y., & Zhang, J. (2010). Access to farming pesticides and risk for suicide in Chinese rural young people. *Psychiatry Research, 179*, 217-221.

Lari, A. R., Alaghehbandan, R., Panjeshahin, M.-R., & Joghataei, M.-T. (2009). Suicidal behavior by burns in the province of Fars, Iran. *Crisis: Journal of Crisis Intervention & Suicide*, *30*, 98-101.

Lari, A. R., Joghataei, M. T., Adli, Y. R., Zadeh, Y. A., & Alaghehbandan, R. (2007). Epidemiology of suicide by burns in the province of Isfahan, Iran. *Journal of Burn Care & Research, 28*, 307-311.

Ma, X., Xiang, T. Y., Cai, Z. J., Li, S. R., Xiang, Y. Q., Guo, H. L., et al. (2009). Lifetime prevalence of suicidal ideation, suicide plans and attempts in rural and urban regions of Beijing, China. *Australian and New Zealand Journal of Psychiatry, 43*, 158-166.

Manoranjitham, S. D., Rajkumar, A. P., Thangadurai, P., Prasad, J., Jayakaran, R., & Jacob, K. S. (2010). Risk factors for suicide in rural south India. *British Journal of Psychiatry, 196*, 26-30.

Manuel, C., Gunnell, D. J., Hoek, W. D., Dawson, A., Wijeratne, I. K., & Konradsen, F. (2008). Self-poisoning in rural Sri Lanka: small-area variations in incidence. *BMC Public Health, 8*.

Mashreky, S. R., Rahman, F., & Rahman, A. (2013). Suicide kills more than 10,000 people every year in Bangladesh. *Archives of Suicide Research,17*(4), 387-396.

Mohanty, B. B. (2005). 'We are like the living dead': Farmer suicides in Maharashtra, western India. *Journal of Peasant Studies, 32*, 243-276.

Mohanty, S., Sahu, G., Mohanty, M. K., & Patnaik, M. (2007). Suicide in India - A four year retrospective study. *Journal of Forensic and Legal Medicine, 14*, 185-189.

Moniruzzaman, S., & Andersson, R. (2008). Cross-national injury mortality differentials by income level: The possible role of age and ageing. *Public Health, 122*, 1167-1176.

Moosa, M. Y. H., Jeenah, F. Y., & Vorster, M. (2005). Repeat non-fatal suicidal behaviour at Johannesburg Hospital. *South African Journal of Psychiatry, 11*, 84-88.

Mukhopadhyay, D. K., Mukhopadhyay, S., Sinhababu, A., & Biswas, A. B. (2012). Are the adolescent behaviors too risky? A school-based study in a District of West Bengal, India. *Journal of Tropical Pediatrics,* 58, 496-500.

Nagthan, S., Rajendra, P., Kunnal, L. B., Basvaraja, H., & Basavaraj, B. (2011). A probe into socio-economic and psychological profile of farmers' suicide in Karnataka. *Karnataka Journal of Agricultural Sciences,* 24, 157-160.

Naidoo, S. C. S., & Schlebusch, L. (2013). Sociodemographic and clinical profiles of suicidal patients requiring admission to hospitals south of Durban. *South African Family Practice, 55*, 373-379.

Nath, Y., Paris, J., Thombs, B., & Kirmayer, L. (2012). Prevalence and social determinants of suicidal behaviours among college youth in India. *International Journal of Social Psychiatry, 58*, 393-399.

Nojomi, M., Bolhari, J., Malakouti, S. K., Shooshtari, M. H., Amin, S. A., & Mashhadi, M. P. (2006). The study of demographic characteristics of suicide attempters attending emergency rooms of Karaj Hospitals in 2003-2004 [Farsi]. *Journal of Iran University of Medical Sciences, 13*, 234-234.

Nojomi, M., Malakouti, S. K., Bolhari, J., Hakimshooshtari, M., Fleischmann, A., & Bertoloted, J. M. (2008). Epidemiology of suicide attempters resorting to emergency departments in Karaj, Iran, 2003. *European Journal of Emergency Medicine, 15*, 221-223.

Nojomi, M., Malakouti, S. K., Bolhari, J., & Poshtmashhadi, M. (2007). A predictor model for suicide attempt: evidence from a population-based study. *Archives of Iranian Medicine, 10*, 452-458.

Ovuga, E., Boardman, J., & Wassermann, D. Y. (2005). Prevalence of suicide ideation in two districts of Uganda. *Archives of Suicide Research, 9*(4), 321-332.

Pandey, M. K., & Kaur, C. (2009). *Investigating Suicidal Trend and its Economic Determinants: Evidence from India.* [ASARC Working Papers](http://econpapers.repec.org/paper/pasasarcc/) from [The Australian National University, Australia South Asia Research Centre](https://crawford.anu.edu.au/acde/asarc/).

Parkar, S. R., Nagarsekar, B. B., & Weiss, M. G. (2012). Explaining suicide: Identifying common themes and diverse perspectives in an urban Mumbai slum. *Social Science & Medicine, 75*, 2037-2046.

Pawan, T., Mohan, R. S., Kalita, R., & Nitin, D. (2012). Study of suicidal poisoning cases in Tertiary Care Hospital in Central India. *Medico-Legal Update, 12*, 96-98.

Polat√∂z, ñ., Kugu, N., Dogan, O., & Aky√ºz, G. (2011). The prevalence of suicidal behaviour and its correlation with certain sociodemographic variables in Sivas province [Turkish]. *Dusunen Adam: Journal of Psychiatry & Neurological Sciences, 24*, 13-23.

Qaisar, F., Mohsin, S., Ayesha, M., Dileep, K., Awais, M., & Umer, M. (2014). The epidemiology of deliberate self-poisoning presenting at a Tertiary Care Hospital in Hyderabad Sindh, Pakistan. *British Journal of Medicine and Medical Research, 4*, 1041-1048.

Ramim, T., Mobayen, M., Shoar, N., Naderan, M., & Shoar, S. (2013). Burnt wives in Tehran: A warm tragedy of self-injury. *International Journal of Burns and Trauma, 3*, 66-71.

Sabzghabaee, A., Soleimani, M., Farajzadegan, Z., Hosseinpoor, S., Mirhosseini, S. M., & Eizadi-Mood, N. (2013). Social risk factors and outcome analysis of poisoning in an Iranian referral medical center: A toxico-epidemiological approach. *Journal of Research in Pharmacy Practice, 2*, 151-155.

Saddichha, S., Vibha, P., Saxena, M. K., & Methuku, M. (2010). Behavioral emergencies in India: a population based epidemiological study. *Social psychiatry and psychiatric epidemiology, 45*, 589-593.

Sadr, S., Seghatoleslam, T., Habil, H., Zahiroddin, A., Bejanzadeh, S., Seghatoleslam, N., et al. (2013). Risk factors for multiple suicide attempts: A critical appraisal of Iranian psychology. *International Medical Journal, 20*, 418-422.

Sauvaget, C., Ramadas, K., Fayette, J. M., Thomas, G., Thara, S., & Sankaranarayanan, R. (2009). Completed suicide in adults of rural Kerala: Rates and determinants. *National Medical Journal of India, 22*, 228-233.

Singh, D. (2009). Profile of intentional deaths in chandigarh zone of india-A 30 years autopsy experience. *International Journal of Medical Toxicology and Legal Medicine, 11*, 8-15.

Stevovic, L. I., Jasovic-Gasic, M., Vukovic, O., Pekovic, M., & Terzic, N. (2011). Gender differences in relation to suicides committed in the capital of Montenegro (Podgorica) in the period 2000-2006. *Psychiatria Danubina,* 23*,* 45-52.

Tahir, M. N., Akbar, A. H., Naseer, R., Khan, Q. O., Khan, F., & Yaqub, I. (2013). Suicide and attempted suicide trends in Mianwali, Pakistan: Social perspective Tendances des suicides et des tentatives de suicide a Mianwali (Pakistan)degree: Perspective sociale. *Eastern Mediterranean Health Journal, 19*, S111-S114.

Tahir, S. M., Memon, A. R., Kumar, M., & Ali, S. A. (2010). Self inflicted burn; a high tide. *Journal of the Pakistan Medical Association, 60*, 338-341.

Thanh, H. T. T., Trung, N. T., Jiang, G. X., Leenaars, A., & Wasserman, D. (2006). Life time suicidal thoughts in an urban community in Hanoi, Vietnam. *BMC Public Health*, *6*.

Toprak, S., Cetin, I., Guven, T., Can, G., & Demircan, C. (2011). Self-harm, suicidal ideation and suicide attempts among college students. *Psychiatry Research, 187*, 140-144.

Toros, F., Bilgin, N. G., Sasmaz, T., Bugdayci, R., & Camdeviren, H. (2004). Suicide attempts and risk factors among children and adolescents. *Yonsei Medical Journal, 45*, 367-374.

Wan, Y.-H., Hu, C.-L., Hao, J.-H., Sun, Y., & Tao, F.-B. (2011). Deliberate self-harm behaviors in Chinese adolescents and young adults. *European Child & Adolescent Psychiatry, 20*, 517-525.

Xie, L.-F., Chen, P.-L., Pan, H.-F., Tao, J.-H., Li, X.-P., Zhang, Y.-J., et al. (2012). Prevalence and correlates of suicidal ideation in SLE inpatients: Chinese experience. *Rheumatology International, 32*, 2707-2714.

Yur'yev, A., Vaernik, A., Vaernik, P., Sisask, M., & Leppik, L. (2012). Employment status influences suicide mortality in Europe. *International Journal of Social Psychiatry, 58*(1), 62-68.

Zaheer, M. S., Aslam, M., Vibhanshu, G., Vibhor, S., & Khan, S. A. (2009). Profile of poisoning cases at a North Indian tertiary care hospital. *Health and Population Perspectives and Issues, 32*, 176-183.

Zhang, J., Jia, S. H., Jiang, C., & Sun, J. (2006). Characteristics of Chinese suicide attempters: An emergency room study. *Death Studies, 30*, 259-268.

Zhang, J., Ma, J., Jia, C., Sun, J., Guo, X., Xu, A., et al. (2010). Economic growth and suicide rate changes: A case in China from 1982 to 2005. *European Psychiatry, 25*, 159-163.

Zhang, J., & Zhou, L. (2009). A case control study of suicides in China with and without mental disorder. *Crisis: The Journal of Crisis Intervention & Suicide Prevention, 30*, 68-72.
